# Demethylase-independent roles of LSD1 in regulating enhancers and cell fate transition

**DOI:** 10.1038/s41467-023-40606-1

**Published:** 2023-08-22

**Authors:** Cheng Zeng, Jiwei Chen, Emmalee W. Cooke, Arijita Subuddhi, Eliana T. Roodman, Fei Xavier Chen, Kaixiang Cao

**Affiliations:** 1 Department of Biochemistry, School of Medicine, Case Western Reserve University, 10900 Euclid Avenue, Cleveland, OH 44106 USA; 2 Case Comprehensive Cancer Center, Case Western Reserve University, 10900 Euclid Avenue, Cleveland, OH 44106 USA; 3 Fudan University Shanghai Cancer Center, Institutes of Biomedical Sciences, Shanghai, China

**Keywords:** Epigenetics, Embryonic stem cells

## Abstract

The major enhancer regulator lysine-specific histone demethylase 1A (LSD1) is required for mammalian embryogenesis and is implicated in human congenital diseases and multiple types of cancer; however, the underlying mechanisms remain enigmatic. Here, we dissect the role of LSD1 and its demethylase activity in gene regulation and cell fate transition. Surprisingly, the catalytic inactivation of LSD1 has a mild impact on gene expression and cellular differentiation whereas the loss of LSD1 protein de-represses enhancers globally and impairs cell fate transition. LSD1 deletion increases H3K27ac levels and P300 occupancy at LSD1-targeted enhancers. The gain of H3K27ac catalyzed by P300/CBP, not the loss of CoREST complex components from chromatin, contributes to the transcription de-repression of LSD1 targets and differentiation defects caused by LSD1 loss. Together, our study demonstrates a demethylase-independent role of LSD1 in regulating enhancers and cell fate transition, providing insight into treating diseases driven by LSD1 mutations and misregulation.

## Abstract (web summary)

Here the authors describe the catalytic-independent role of LSD1 in regulating gene expression and cellular differentiation and a mechanism by which the LSD1-P300 axis contributes to the regulation of enhancers and cell fate.

## Introduction

Enhancer malfunction is a key driver of human cancers and congenital disorders such as Kabuki syndrome, Cornelia de Lange syndrome, and Rubinstein-Taybi syndrome^1–3^. The activity of enhancers could be regulated by the enhancer’s affinity to transcription factors, the balance between co-activators and co-repressors, and local chromatin architecture. In mammals, enhancers are decorated by nucleosomes harboring mono-methylated lysine 4 of histone H3 (H3K4me1)^4^, which is deposited by methyltransferases MLL3 and MLL4 and removed by lysine specific demethylase 1 (LSD1)^5–9^. We and others have recently demonstrated that the catalytic activity of MLL3 and MLL4 is dispensable for transcriptional regulation, cell fate transition, and animal development^6,10,11^, indicating that H3K4me1 at enhancers does not have a major impact on gene regulation. Nevertheless, the antagonism between MLL4 and LSD1 on enhancers plays a critical role in modulating enhancer activity and cellular differentiation^6^, suggesting potential catalytic-independent roles of LSD1 in enhancer regulation. Despite being heavily studied, molecular mechanisms underlying the roles of epigenetic modifiers in regulating enhancers, transcription, and cell fate remain elusive.

LSD1 is the first identified histone lysine demethylase, targeting enhancers and removing H3K4me1/2 via its flavin adenine dinucleotide (FAD)-dependent amine oxidase activity^7–9,12^. LSD1 is a component of the transcriptional co-repressor complex CoREST, which contains the adaptor protein RCOR1 and histone deacetylases HDAC1 and HDAC2^9,13,14^. RCOR1 directly interacts with HDAC1/2 and LSD1, bridging the two enzymatic moieties into one protein complex^13,15^. Such a dual enzymatic role of CoREST is believed to be essential for gene repression^14,16^. In addition to its importance in embryogenesis and differentiation^7–9,12,17–19^, LSD1 is implicated in developmental disorders, inflammatory diseases, neurodegenerative diseases, and cancer^20–26^, rendering it an attractive therapeutic target. As a result, various inhibitors of LSD1 have been developed and are currently undergoing clinical trials^27^. Nonetheless, several lines of evidence suggest that LSD1 could function independently from its demethylase activity^28–31^, raising an important question on how LSD1 catalytically and non-catalytically regulates gene expression.

Pluripotent stem cell (PSC) models including embryonic stem cells (ESCs) are ideal to understand the mechanisms underlying mammalian development because of their capability of differentiating into almost all cell types in adult tissues. It is well established that LSD1 is required for PSC differentiation^18,19^; however, the role of LSD1 in lineage specification is not well understood. Moreover, previous studies of LSD1 have focused on its demethylase activity, the function of which has not been genetically examined. We therefore aimed to unveil the mechanisms by which LSD1 regulates cellular differentiation using ESCs as the model in this study.

Here, we dissected the catalytic and non-catalytic function of LSD1 by mutating the endogenous LSD1 to inactivate its demethylase activity and delete LSD1 in ESCs, respectively. To our surprise, LSD1 functions mainly in a catalytic-independent manner in regulating gene expression and cellular differentiation. Moreover, LSD1 is not required for the recruitment of the CoREST complex to chromatin despite the destabilization of CoREST components RCOR2 and RCOR1 in LSD1 null cells. Furthermore, the balance between LSD1 and acetyltransferases P300/CBP at enhancers is critical for the regulation of LSD1 target genes and cell fate transition. Our results not only elucidate how LSD1 regulates enhancer activity, transcriptional outputs, and cell identity, but also provide alternative strategies to better target diseases driven by LSD1 loss- and gain-of-function.

## Results

### Demethylase-independent roles of LSD1 in gene regulation

**Fig. 1:**
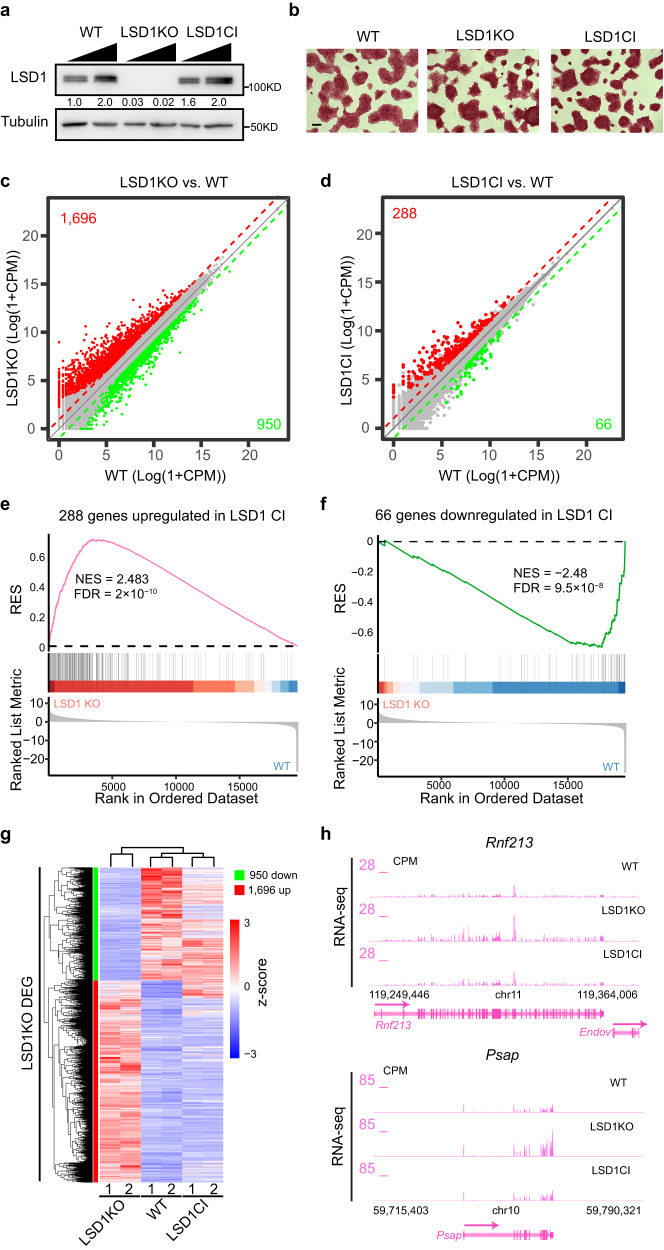
Catalytic-independent roles of LSD1 in gene regulation. **a** Western blotting indicating LSD1 levels in WT, LSD1 KO, and LSD1 CI ESCs. 15 and 30 μg proteins from total cell lysates were loaded for each sample. LSD1 levels in each lane were quantified by normalizing with Tubulin signals of the corresponding lane. Normalized ratios were provided under the LSD1 blot. This experiment was repeated three times independently with similar results observed. Source data are provided as a Source data file. **b** Alkaline phosphatase staining of WT, LSD1 KO, and LSD1 CI cells. This experiment was repeated three times independently with similar results observed. Scale bar: 100 μm. **c**, **d** Correlation plots of RNA-seq data between LSD1 KO (**c**) or LSD1 CI (**d**) cells and WT cells. RNA-seq experiments were performed with two biological replicates from WT ESCs and two independent mutant cell clones. Statistical significance was determined by two-sided Wald test and *p*-values were corrected for multiple testing using the Benjamini–Hochberg method. Significantly upregulated genes (log2 fold change >1, adjusted *p* < 0.01) were highlighted in red while downregulated genes (log2 fold change <−1, adjusted *p* < 0.01) were highlighted in green. The number of up- and downregulated genes are listed on the plots. **e**, **f** GSEA analysis of genes upregulated (**e**) and downregulated (**f**) in LSD1 CI ESCs comparing LSD1 KO and WT ESCs. RES: running enrichment score; NES: normalized enrichment score; FDR: false discovery rate. **g** Hierarchical clustering analysis of expression levels of the 2646 differentially regulated genes in LSD1 KO cells comparing WT, LSD1 KO, and LSD1 CI ESCs. Z-scores were used to generate the heatmap. Numbers below the heatmap denote the 2 biological replicates of each genotype. **h** Genome browser view of RNA-seq signals at representative LSD1 target genes in WT and LSD1 mutant ESCs. CPM: counts per million mapped reads.

We previously demonstrated the antagonism between H3K4me1 regulators MLL4 and LSD1 in ESCs^6^. The catalytic activity of MLL4 is dispensable for enhancer activation and cell fate transition^6,10,11^; however, whether the catalytic activity of LSD1 plays a role in these processes remains unknown. To investigate the role of LSD1 in gene regulation, we first generated LSD1 knockout (KO) ESCs by deleting the promoter and the first exon of *Lsd1* gene using CRISPR/Cas9 (Supplementary Fig. 1a). The resulting loss of LSD1 protein was confirmed by Western blotting (Supplementary Fig. 1b). We further mutated the key catalytic residues lysine 661 (K661)^32^ and alanine 539 (A539)^33^ of the endogenous LSD1 to glutamine (Q) and glutamic acid (E), respectively, in ESCs via CRISPR/Cas9 to study the demethylase function of LSD1 (Supplementary Fig. 1c, e). It is noteworthy that we attempted to generate LSD1 K661A mutant alleles; however, the K661A mutation resulted in decreased LSD1 levels compared with WT cells, possibly because the mutation in the AG consensus sequence at the 3’ of exon 16 disrupts proper splicing of the *Lsd1* gene (Supplementary Fig. 1c, d). On the other hand, the LSD1 K661Q mutation had little impact on LSD1 protein levels. The levels of LSD1 in ESCs harboring both the K661Q and A539E mutations were comparable to wildtype (WT) cells (Fig. 1a), indicating that the level of the catalytic inactive (CI) LSD1 is comparable to its WT counterpart. We used the LSD1 K661Q/A539E mutant ESCs as the CI mutant in our study since simultaneously mutating the A539 and K661 residues completely abolishes the demethylase activity of LSD1 in vitro^33^. LSD1 KO and CI ESCs were morphologically comparable to their WT counterpart and alkaline phosphatase (AP) positive (Fig. 1b). Moreover, both WT and K661Q/A539E mutated LSD1 interacted with core components of the CoREST complex (Supplementary Fig. 1f). Furthermore, WT, LSD1 KO, and LSD1 CI ESCs had similar expression levels of pluripotency genes *Pou5f1* and *Sox2* (Supplementary Fig. 1g). Taken together, these data indicated that LSD1 and its demethylase activity are not required for the maintenance of ESC self-renewal.

RNA-seq analysis from two independent cell clones indicated that LSD1 deletion in ESCs led to the misregulation of 2,646 genes (Fig. 1c). In contrast, LSD1 inactivation caused the deregulation of 354 genes (Fig. 1d), indicating a milder impact of LSD1 catalytic inactivation on gene expression. Among 288 significantly upregulated genes in LSD1 CI cells, 158 genes were significantly upregulated by LSD1 deletion (Supplementary Fig. 1h). To understand the functional relationship between LSD1 deletion and inactivation on gene regulation, we compared RNA-seq data from LSD1 KO and WT ESCs and performed gene set enrichment analysis (GSEA) using the 288 upregulated genes and 66 downregulated genes in LSD1 CI cells as gene lists. Genes upregulated by LSD1 inactivation were significantly upregulated by LSD1 deletion and genes downregulated by LSD1 inactivation were significantly downregulated in LSD1 KO cells (Fig. 1e, f). Hierarchical clustering analysis and visualization of RNA-seq data further revealed the difference between the effects of LSD1 deletion compared with that of catalytic inactivation on gene expression (Fig. 1g, h, Supplementary Fig. 1i). To further investigate how LSD1 antagonizes MLL4 in regulating transcription, we generated MLL4 and LSD1 double KO (DKO) and MLL4KO/LSD1CI cells by deleting MLL4 in LSD1 KO and LSD1 CI cells, respectively (Supplementary Fig. 1j, k). Interestingly, LSD1 deletion but not catalytic inactivation markedly rescued expression levels of genes misregulated by MLL4 deletion (Supplementary Fig. 1l, m). In summary, our results demonstrated that LSD1 regulates gene expression in ESCs mainly in a demethylase-independent manner.

### LSD1 deletion rather than inactivation leads to enhancer de-repression

**Fig. 2:**
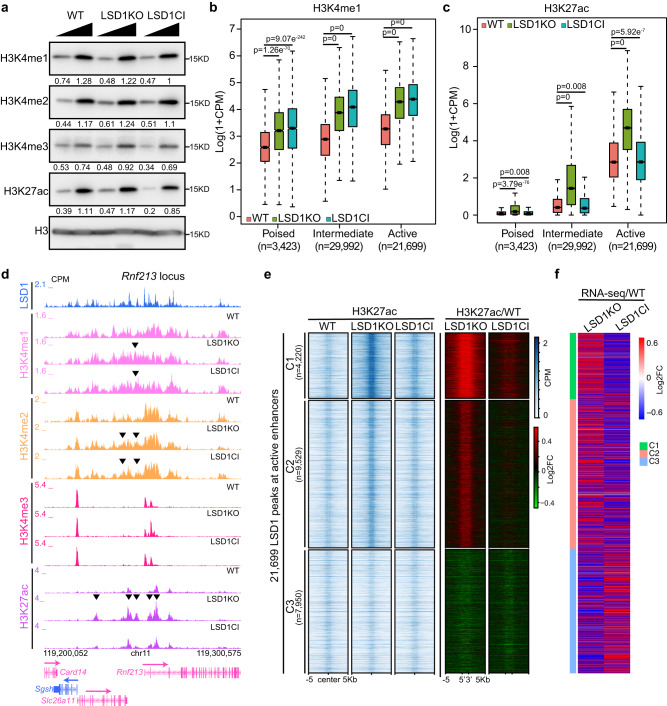
LSD1 deletion rather than catalytic inactivation leads to enhancer de-repression. **a** Western blotting of H3K4me1/2/3 and H3K27ac in WT, LSD1 KO, and LSD1 CI cells. Levels of histone modifications in each lane were quantified by normalizing with H3 signals of the corresponding lane. Normalized ratios were provided under each histone modification blot. This experiment was repeated three times independently with similar results observed. Source data are provided as a Source data file. **b**, **c** Box plots of H3K4me1 (**b**) and H3K27ac (**c**) levels at LSD1-bound enhancers in WT, LSD1 KO, and LSD1 CI cells. 3423 poised, 29,992 intermediate, and 21,699 active enhancers were called based on H3K4me1, H3K27ac, H3K27me3, and LSD1 ChIP-Rx data. *n* = 2 biologically independent experiments. *P*-values (*p*) from two-sided Wilcoxon signed-rank tests on log2(LSD1KO/WT) and log2(LSD1CI/WT) are denoted in each panel. Center line: median; top and bottom hinges of box: the third and first quantiles; whiskers: quartiles ± 1.5 × interquartile range. **d** Genome browser view of H3K4me1/2/3 and H3K27ac ChIP-Rx signals at the *Rnf213* locus in WT, LSD1 KO, and LSD1 CI ESCs. Black arrows indicate changes between LSD1 mutant and WT cells. **e** Heat maps showing H3K27ac ChIP-Rx levels at LSD1-enriched active enhancers in WT, LSD1 KO, and LSD1 CI ESCs. Log2 fold change between LSD1 mutant and WT cells are shown on the right. Clusters were generated by k-means clustering, and signals 5 kb up- and downstream of LSD1 peak regions were included. The number of peaks under each cluster was labeled in parentheses. **f** Heat maps showing the log2 fold change of RNA-seq signals between LSD1 mutant and WT ESCs. The nearest genes to each LSD1 enriched active enhancer were used to generate the heat map. Clusters are the same as in (**e**).

To understand the mechanisms underlying the demethylase-independent role of LSD1, we measured levels of H3K4 methylation (H3K4me) and the active enhancer mark H3K27ac in WT and LSD1 mutants. Western blotting indicated that neither LSD1 deletion nor catalytic inactivation changed global levels of H3K4me or H3K27ac (Fig. 2a). Such results were consistent with a recent report demonstrating that LSD1 deletion has little impact on global levels of H3K4me^34^. To determine if local levels of H3K4me and H3K27ac are modulated by LSD1 and its demethylase activity, we performed ChIP with reference exogenous genome (ChIP-Rx) of H3K4me1/2/3 and H3K27ac in WT, LSD1 KO, and LSD1 CI ESCs. H3K4me1 enriched and LSD1-bound non-TSS (transcription start site) regions were grouped into poised (H3K4me1/H3K27me3 enriched), active (H3K4me1/H3K27ac enriched), and intermediate (only enriched with H3K4me1) enhancers^4,35,36^. LSD1 deletion and catalytic inactivation led to an increase of H3K4me1 and H3K4me2 at all three enhancer groups (Fig. 2b, d, Supplementary Fig. 2a, c), indicating that the demethylase activity of LSD1 is required for modulating enhancer H3K4 methylation. On the other hand, LSD1 deletion but not catalytic inactivation led to a minor increase of H3K4me3 levels at active and intermediate enhancers (Supplementary Fig. 2b), corroborating prior results that LSD1 demethylates H3K4me1 and H3K4me2 in vivo^12^. The increase of H3K4me3 levels at enhancers in LSD1 null cells may reflect the enhancer de-repression caused by the loss of LSD1. Interestingly, the levels of H3K27ac at enhancers were elevated in LSD1 KO but not CI ESCs (Fig. 2c, d, Supplementary Fig. 2c), suggesting that the demethylase activity of LSD1 is dispensable for enhancer decommissioning. To understand the relationship between the increase of H3K27ac levels and gene expression, we compared H3K27ac levels in WT and LSD1 mutant cells at LSD1-bound active enhancers by k-means clustering. Our results showed that H3K27ac levels at two out of three clusters of LSD1-bound active enhancers are significantly elevated in LSD1 KO ESCs and that the increase of H3K27ac is correlated with de-repression of genes near these enhancers (Fig. 2e, f, Supplementary Fig. 2d, e). Overall, these data indicated that LSD1 regulates enhancer activity independently from its demethylase activity.

### LSD1 deletion rather than inactivation impairs ESC differentiation

Although LSD1 plays important roles in regulating embryogenesis and stem cell differentiation^17–19^, the function of LSD1’s demethylase activity in cellular differentiation remains elusive. To answer this question, we performed spontaneous differentiation, epiblast-like cell (EpiLC) differentiation, and embryoid body (EB) differentiation using WT, LSD1 KO, and LSD1 CI ESCs. For spontaneous differentiation, we grew ESCs in serum containing media without supplementing the MEK inhibitor and GSK inhibitor (2i) or leukemia inhibitory factor (LIF) for four days. After spontaneous differentiation, LSD1 KO cells had a higher level of alkaline phosphatase compared with WT and LSD1 CI cells (Supplementary Fig. 3a), suggesting that LSD1 deletion prohibits ESC differentiation while LSD1 catalytic inactivation has little impact. RNA-seq analysis indicated that genes typically downregulated during spontaneous differentiation, such as the naive pluripotency marker *Esrrb*, are upregulated in LSD1 KO cells upon differentiation; however, such a trend was not observed in LSD1 CI cells (Supplementary Fig. 3b). For EpiLC differentiation, naive ESCs were cultured in media containing FGF2 and activin A for 2 days^37^. LSD1 deletion rather than inactivation led to an increase of naive pluripotency marker *Klf2* during EpiLC differentiation (Supplementary Fig. 3c), suggesting that LSD1 deletion but not catalytic inactivation impairs ESCs from exiting naive pluripotency.

**Fig. 3:**
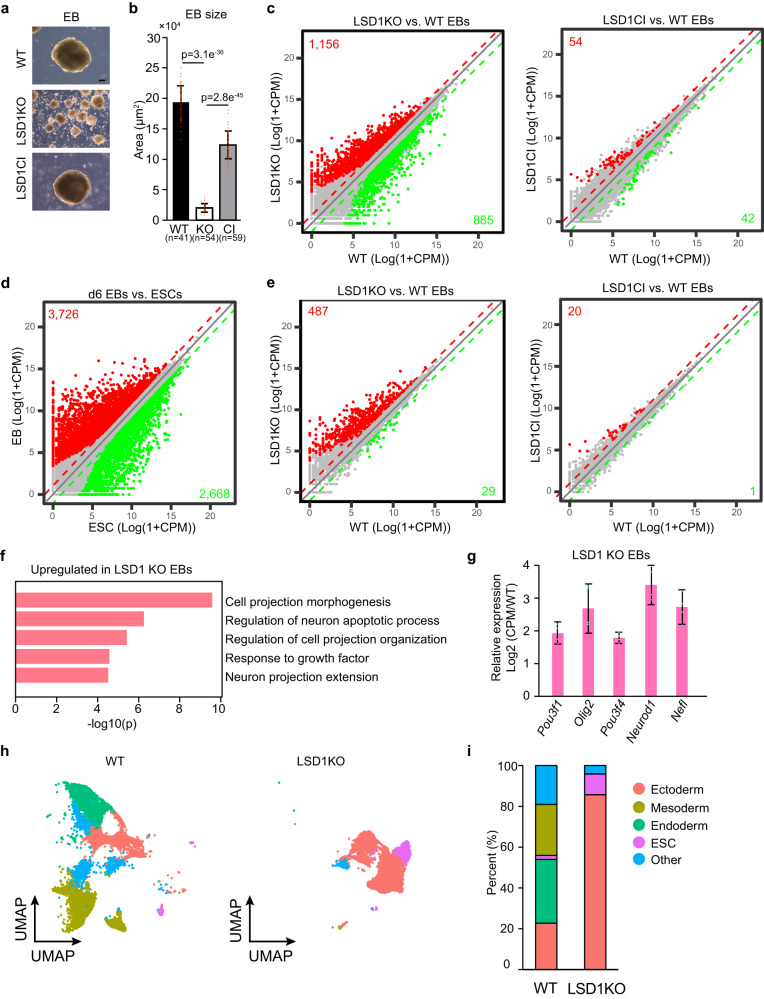
Catalytic-independent roles of LSD1 in cellular differentiation. **a** Phase-contrast images of day 6 EBs generated from WT, LSD1 KO, and LSD1 CI ESCs. Scale bar: 100 μm. Experiments were repeated three times independently with similar results observed. **b** Quantification of EB sizes in (**a**). Data are presented as mean values ± standard deviation (SD). *n* = 3 biologically independent experiments. *P*-values were calculated using two-sided student’s t-test. Source data are provided as a Source data file. **c** Correlation plots of RNA-seq data between LSD1 KO (left) or CI (right) and WT day 6 EBs. Data are derived from two biological replicates from two cell clones. Statistical significance was determined by two-sided Wald test and *p*-values were corrected for multiple testing using the Benjamini–Hochberg method. Significantly up- and downregulated genes are labeled in red and green with numbers of genes noted, respectively. **d** Correlation plots of RNA-seq data between day 6 EBs and ESCs. Data are derived from two biological replicates. Statistical significance was determined by two-sided Wald test and *p*-values were corrected for multiple testing using the Benjamini–Hochberg method. **e** Correlation plots of RNA-seq data as in (**c**) with downregulated genes in day 6 EBs vs. ESCs (2668 green genes in **d**) shown. Data are derived from two biological replicates. Statistical significance was determined by two-sided Wald test and *p*-values were corrected for multiple testing using the Benjamini–Hochberg method. Significantly up- and downregulated genes are labeled in red and green with numbers of genes noted, respectively. **f** Gene ontology (GO) analysis of genes upregulated in LSD1 KO day 6 EBs. Top 5 GO terms are shown. **g** Fold change of RNA-seq signals (CPM) in LSD1 KO over WT EBs is shown for neural marker genes. Data are presented as mean values ± SD. *n* = 2 biologically independent experiments. Source data are provided as a Source data file. **h** scRNA-seq gene expression projected onto a UMAP space for day 6 WT and LSD1 KO EBs. Inferred cell types based on marker genes were highlighted. Data were derived from two biological replicates. **i** Quantification of cell types in day 6 WT and LSD1 KO EBs based on scRNA-seq data.

Consistent with published results^18,19^, we observed impaired EB differentiation in LSD1 KO ESCs, while the morphology of LSD1 CI EBs was comparable to that of WT EBs (Fig. 3a, b). Furthermore, approximately 2000 genes were misregulated in LSD1 KO EBs, while only about 100 genes were deregulated in LSD1 CI EBs (Fig. 3c). Notably, ~18% of genes downregulated during EB differentiation were significantly de-repressed after LSD1 deletion, compared with ~0.7% of those genes significantly upregulated upon LSD1 inactivation (Fig. 3d, e). Moreover, hierarchical clustering and box plot analyses of RNA-seq data demonstrated that genes downregulated during EB differentiation have lower expression levels in WT and LSD1 CI EBs than that in LSD1 KO EBs (Supplementary Fig. 3d, e). These data indicated that LSD1 plays a catalytic-independent role in regulating cellular differentiation. To further understand the impact of LSD1 loss on gene regulation during differentiation, we performed gene ontology (GO) analysis of up- and downregulated genes in LSD1 null EBs compared with WT EBs. Interestingly, neural related genes were significantly enriched in LSD1 suppressed genes during differentiation (Fig. 3f, g). Moreover, ChIP-Rx analysis revealed increased H3K4me1 and H3K27ac levels at neural marker genes in LSD1 KO EBs (Supplementary Fig. 3f), indicating that the epigenetic reprogramming upon LSD1 loss is correlated with the gain of neural gene expression during differentiation. In contrast, genes related to heart development were highly enriched in downregulated genes of LSD1 null EBs (Supplementary Fig. 3g). We noted that cardiac marker genes were downregulated in LSD1 CI EBs although such downregulation was not as drastic as seen in LSD1 KO EBs (Supplementary Fig. 3h). To further understand the role of LSD1 on cellular trajectories during differentiation, we performed single cell RNA-seq (scRNA-seq) on WT and LSD1 KO day 6 EBs. Our analysis indicated that LSD1 deletion leads to the enrichment of ectodermal cells and the depletion of mesodermal and endodermal cells in EBs (Fig. 3h, i), consistent with bulk RNA-seq results that neural genes were upregulated and that cardiac genes were downregulated in LSD1 null EBs. To understand if LSD1 loss or inactivation plays any role in cardiac differentiation, we differentiated WT and LSD1 mutant ESCs into cardiomyocytes using a well-established protocol^38^ (Supplementary Fig. 3i). We found that LSD1 deletion leads to smaller mesodermal EBs in day 4 compared with WT cultures (Supplementary Fig. 3j). Moreover, LSD1 KO cultures did not generate any cardiac precursors or cardiomyocytes (Supplementary Fig. 3j). Despite having smaller mesodermal EBs, LSD1 CI cultures were able to generate cardiac Troponin T (cTnT) positive cardiomyocytes with a less robust cardiomyocyte network than their WT counterparts (Supplementary Fig. 3j, k), suggesting that LSD1 is required for the cardiac lineage commitment while its demethylase activity fine-tunes the process. Taken together, our data obtained from four distinct differentiation strategies demonstrated that the demethylase activity of LSD1 is largely dispensable for cell fate transition while the full-length LSD1 protein is required.

### Interdependency of LSD1 and RCOR1/2 in regulating gene expression and cellular differentiation

**Fig. 4:**
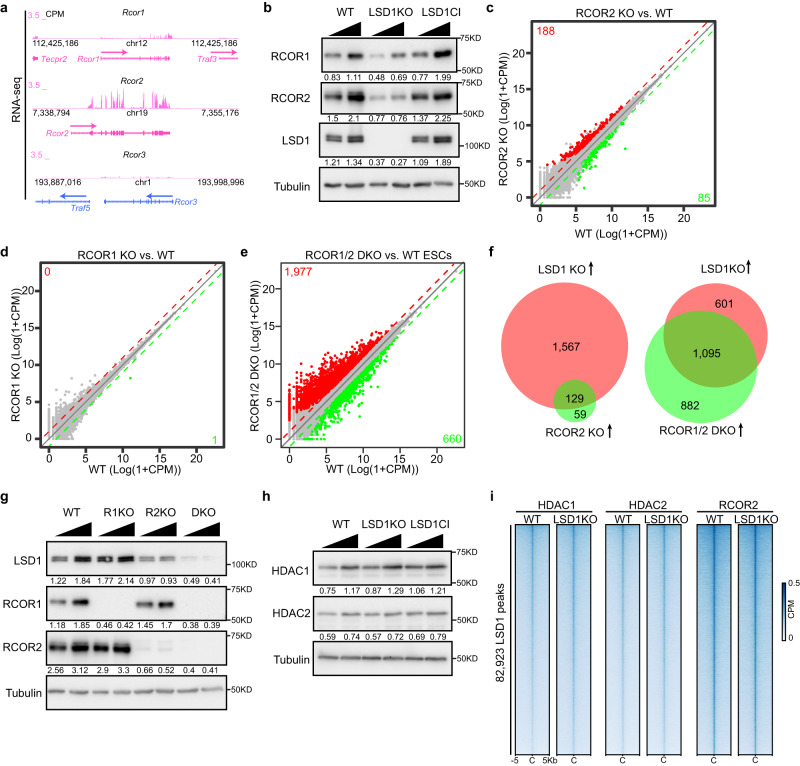
Interdependency of LSD1 and RCORs in regulating gene expression. **a** Genome browser view of RNA-seq signals at *Rcor1*, *Rcor2*, and *Rcor3* genes in ESCs. (**b**) Western blotting indicating the level of RCOR1, RCOR2, LSD1, and Tubulin in WT, LSD1KO, and LSD1CI ESCs. Levels of RCORs and LSD1 in each lane were quantified by normalizing with Tubulin signals of the corresponding lane. Normalized ratios were provided under each blot. Experiments were repeated three times independently with similar results observed. Source data are provided as a Source data file. **c**–**e** Correlation plot of RNA-seq data between RCOR2 KO (**c**), RCOR1 KO (**d**), RCOR1/2 DKO (**e**) and WT ESCs. Data are derived from two biological replicates. Statistical significance was determined by two-sided Wald test and p-values were corrected for multiple testing using the Benjamini-Hochberg method. Significantly up- and downregulated genes are labeled in red and green with numbers of genes noted, respectively. **f** Venn diagrams showing the overlap of upregulated genes between LSD1 KO and RCOR2 KO (left) or RCOR1/2 DKO (right) ESCs. **g** Western blotting of LSD1, RCOR1, RCOR2, and Tubulin in WT and RCOR1/2 mutant cells. R1KO: RCOR1 KO; R2KO: RCOR2 KO; DKO: RCOR1/2 DKO. Levels of LSD1 and RCORs in each lane were quantified by normalizing with Tubulin signals of the corresponding lane. Normalized ratios were provided under each blot. Experiments were repeated three times independently with similar results observed. Source data are provided as a Source data file. **h** Western blotting of HDAC1, HDAC2, and Tubulin in WT, LSD1 KO, and LSD1 CI ESCs. Levels of HDAC1 and HDAC2 in each lane were quantified by normalizing with Tubulin signals of the corresponding lane. Normalized ratios were provided under each blot. Experiments were repeated three times independently with similar results observed. Source data are provided as a Source data file. **i** Heat maps showing the ChIP-Rx levels of HDAC1, HDAC2, and RCOR2 at LSD1 enriched regions in WT and LSD1 KO ESCs.

LSD1 is a part of the CoREST complex containing HDAC1/2 and RCOR1^7,9,12–15^. Structural studies indicate that RCOR1 directly binds the nucleosome and interacts with the TOWER domain of LSD1^33,39^. RCOR1 also directly binds HDAC1/2, tethering the demethylase and deacetylase activities in the same complex^13,15,40^. Since LSD1 deletion leads to an increase of H3K27ac at enhancers (Fig. 2), we speculated that LSD1 regulates H3K27ac through HDAC1/2 such that LSD1 loss leads to the dissociation of RCOR1 from chromatin followed by a decreased HDAC1/2 recruitment and an increase in H3K27ac. RCOR1 has two paralogs, RCOR2 and RCOR3, which also associate with LSD1 and HDAC1/2^41–43^. While RCOR3 was minimally expressed in ESCs, the expression level of RCOR2 was much higher (Fig. 4a). Moreover, RCOR2 plays an important role in somatic cell reprogramming to iPSCs and cortical development^41,44^. We therefore examined the level of RCOR1 and RCOR2 in WT and LSD1 mutants. Western blotting demonstrated that RCOR2 level is reduced more than two-fold by LSD1 deletion, while the level of RCOR1 is moderately decreased (Fig. 4b). These data are reminiscent of previous findings that LSD1 depletion has little impact on RCOR1 level in human cells^13^. To understand if LSD1 regulates gene expression and H3K27ac level at enhancers through modulating RCOR2 level, we deleted RCOR2 in ESCs using CRISPR/Cas9. Unexpectedly, we did not observe obvious differences in the morphology and growth rate of WT and RCOR2 KO ESCs. Such observation contrasted with previous reports that RCOR2 depletion by shRNA leads to the impairment in ESC proliferation^41^, possibly because our ESCs were maintained in serum free media at the ground state. RNA-seq analysis indicated that RCOR2 deletion causes the misregulation of 273 genes (Fig. 4c), markedly less than deregulated genes in LSD1 null ESCs. ~69% of RCOR2-repressed genes overlapped with LSD1-repressed genes (Fig. 4f), indicating a functional overlap between RCOR2 and LSD1 in gene repression. Since LSD1 deletion moderately downregulated RCOR1 (Fig. 4b), we deleted RCOR1 using CRISPR/Cas9 to determine if LSD1 regulates gene expression through RCOR1. However, we found that RCOR1 deletion does not significantly perturb the ESC transcriptome (Fig. 4d). Since RCOR1 and RCOR2 have the same functional domains, interact with LSD1 and HDACs, and harbor similar transcriptional repressive capability in vitro^41–43^, it is possible that RCOR1 and RCOR2 are redundant in regulating gene expression in vivo. To examine such a possibility, we generated RCOR1/2 double KO (DKO) ESCs using CRISPR/Cas9. RNA-seq analysis revealed that RCOR1/2 DKO cells harbor 2,637 misregulated genes (Fig. 4e). Furthermore, ~64% of LSD1-repressed genes were de-repressed in RCOR1/2 DKO ESCs (Fig. 4f), suggesting that RCOR1 and RCOR2 redundantly silence LSD1 target genes.

We next investigated the role of RCOR1 and RCOR2 in cellular differentiation. RCOR1 KO and RCOR2 KO cells generated EBs similar in size to WT EBs (Supplementary Fig. 4a, b) despite 1,247 genes being misregulated by RCOR2 deletion during EB differentiation (Supplementary Fig. 4d), indicating that RCOR2 is required for proper gene expression during differentiation. Similar to the undifferentiated state, RCOR1 deletion has little impact on gene expression during EB differentiation (Supplementary Fig. 4c). In contrast, RCOR1/2 DKO EBs were much smaller in size and had 4,355 misregulated genes (Supplementary Fig. 4a, b & e), demonstrating that RCOR1 and RCOR2 redundantly control cellular differentiation. More than 72% genes upregulated in LSD1 null EBs overlapped with elevated genes in RCOR1/2 DKO EBs (Supplementary Fig. 4f), suggesting that LSD1 is functionally related to RCOR1/2 in regulating stem cell differentiation. To understand how RCOR1/2 modulate gene expression and cellular differentiation, we first measured the level of LSD1 in RCOR mutant cells. Western blotting analysis indicated that RCOR2 deletion leads to a decrease in LSD1 level (Fig. 4g), suggesting that the lowered LSD1 level could cause minor defects in gene expression and EB differentiation in RCOR2 KO cells. Furthermore, RCOR1/2 deletion led to a drastically (4-fold) reduced LSD1 level (Fig. 4g), suggesting that the stability of LSD1 and RCOR1/2 is interdependent. Thus, it is possible that LSD1 regulates gene expression and cell fate transition through modulating the levels of CoREST complex components RCORs and HDACs, leading to enhancer decommissioning; however, we found that LSD1 deletion or catalytic inactivation had little impact on the global level of HDAC1 and HDAC2 (Fig. 4h). Moreover, the occupancy of HDAC1, HDAC2, and RCOR2 at LSD1 enriched regions was comparable between WT and LSD1 KO ESCs as shown by ChIP-Rx analysis (Fig. 4i), suggesting that the enhancer de-repression after LSD1 deletion is not due to the loss of CoREST recruitment on chromatin.

To further investigate the functional relationship between LSD1 and RCORs in gene regulation, we generated doxycycline (DOX)-inducible and 3×HA tagged LSD1 expressing ESCs in the RCOR1/2 DKO background (hereby named DKO + LSD1) using a PiggyBac transposase system^37^ (Supplementary Fig. 5a). Western blotting analysis indicated that LSD1 level after DOX induction in DKO + LSD1 cells is comparable to that in WT ESCs (Supplementary Fig. 5b). Upon DOX induction in DKO + LSD1 cells, ChIP-Rx analysis identified 3182 LSD1 peaks and increased LSD1 signals at WT LSD1 binding sites (Supplementary Fig. 5c, g, h), suggesting that LSD1 can be recruited to chromatin to a certain extent in a CoREST-independent manner. However, the number of LSD1 peaks (3182) in DKO + LSD1 null cells upon DOX induction was much lower than that in WT cells (82,923, also compare Supplementary Fig. 5c and Fig. 2d), indicating that RCOR1/2 are critical for fully recruiting LSD1 to chromatin. Interestingly, the commonly upregulated genes in LSD1 KO and RCOR1/2 DKO ESCs were not downregulated upon LSD1 restoration (Supplementary Fig. 5d–f), suggesting that RCOR1/2 are required for LSD1 mediated gene repression. Taken together, our data suggest that LSD1 and RCOR1/2 depend on each other to maintain the stability of CoREST complex, and that RCOR1/2 function redundantly to regulate gene expression, cell fate transition, and the recruitment of LSD1 to chromatin.

### P300/CBP contribute to the deregulation of gene expression and differentiation caused by LSD1 loss

**Fig. 5:**
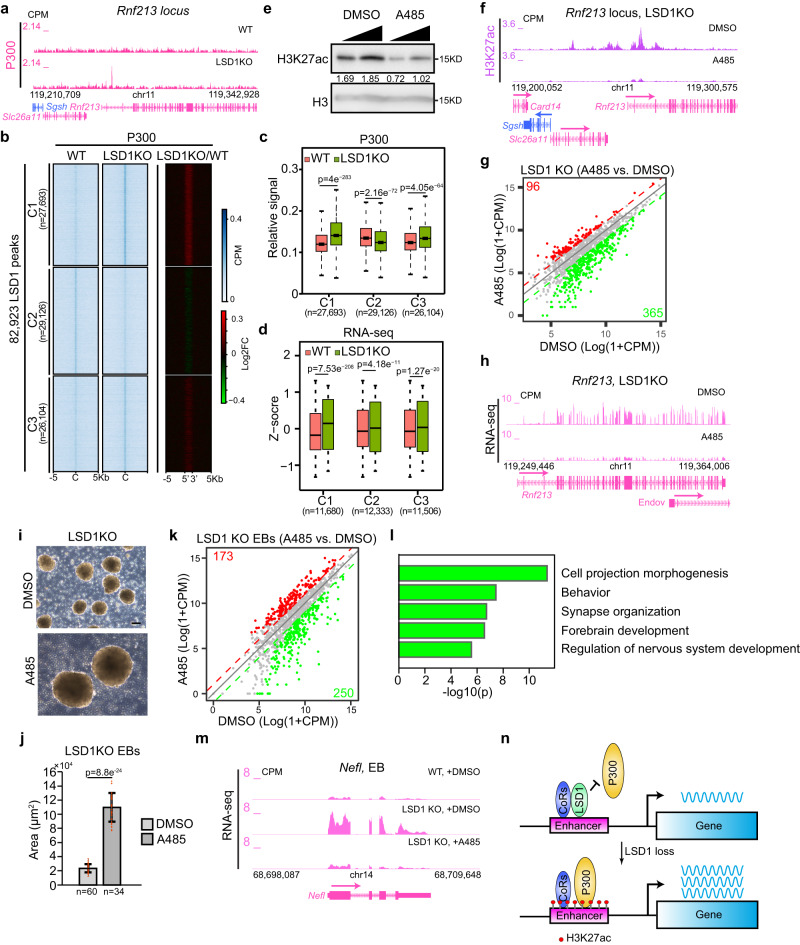
P300/CBP contribute to the gene misregulation and defective differentiation caused by LSD1 loss. **a** Genome browser view of P300 ChIP-Rx signals at *Rnf213* locus in WT and LSD1 KO ESCs. **b** Heatmaps showing P300 ChIP-Rx levels at LSD1 enriched regions in WT and LSD1 KO ESCs. Three clusters were generated by k-means clustering. **c** Box plots indicating the signals of P300 ChIP-Rx in WT and LSD1 KO cells at LSD1 peaks in the three clusters in (**b**). *n* = 2 biologically independent experiments. **d** Box plots of RNA-seq signals of nearest genes to LSD1 peaks in WT and LSD1 KO cells in the three clusters in (**b**). *P*-values in (**c**) and (**d**) were calculated using two-sided Wilcoxon signed-rank tests. Center line: median; top and bottom hinges of box: the third and first quantiles; whiskers: quartiles ± 1.5 × interquartile range. **e** Western blotting of H3K27ac in ESCs treated with DMSO or 10 μM A485 for 24 h. Experiments were repeated three times independently with similar results observed. Source data are provided as a Source data file. **f** Genome browser view of H3K27ac ChIP-Rx signals at *Rnf213* locus in LSD1 KO ESCs treated with DMSO or 10 μM A485 for 24 h. **g** Correlation analysis of upregulated genes upon LSD1 deletion (1696 red genes in Fig. 1c) in A485 *vs*. DMSO treated LSD1 null cells. **h** Genome browser view of RNA-seq signals of *Rnf21*3 gene in LSD1 KO cells treated with DMSO or 10 μM A485 for 24 h. **i** Phase-contrast images of day 6 EBs generated from LSD1KO ESCs treated with DMSO or 0.4 μM A485. Experiments were repeated three times independently with similar results observed. Scale bar: 100 μm. **j** Quantification of EB sizes in (**i**). Data are presented as mean values ± SD. *n* = 3 biologically independent experiments. *P*-values were calculated using two-sided student’s t-test. Source data are provided as a Source data file. **k** Correlation analysis of upregulated genes in LSD1 null EBs (1156 red genes in Fig. 3c) treated with respective 0.4 μM A485 and DMSO. **l** GO analysis of 250 genes upregulated in LSD1 KO EBs but downregulated by A485 treatment (green genes in **k**). **m** Genome browser view of *Nefl* RNA-seq signals in WT and LSD1 KO day 6 EBs treated with DMSO or A485. **n** A working model for the regulation of enhancers by LSD1. P300 occupancy increases at LSD1-targeted enhancers upon LSD1 loss, acetylating nucleosomes at enhancers previously decommissioned by LSD1 and activating gene expression.

In mammals, H3K27ac is catalyzed by acetyltransferases P300/CBP^45^. To understand if LSD1 loss leads to changes in the recruitment of P300/CBP to chromatin, we performed ChIP-Rx and found that the genome occupancy of P300 increases at two out of three clusters of all LSD1 binding sites in LSD1 KO compared with WT ESCs via k-means clustering analysis (Fig. 5a–c). Moreover, the increase in P300 occupancy at LSD1 peaks upon LSD1 deletion was correlated with elevated levels in H3K27ac and gene expression (Fig. 5b–d, Supplementary Fig. 6a, c, d, Fig. 2e, f, and Supplementary Fig. 2d, e), suggesting that the gain of P300 recruitment at enhancers contributes to the H3K27ac increase at enhancers in LSD1 KO ESCs. Interestingly, LSD1 enriched regions in WT cells that gained P300 binding upon LSD1 deletion were enriched with DNA binding motifs of the Krüppel-like factor (KLF) family of transcription factors (Supplementary Fig. 6b), suggesting that KLFs may be responsible for recruiting P300 to enhancers upon LSD1 loss. To further study the relationship between LSD1 and P300 on chromatin recruitment, we expressed 3×HA tagged LSD1 in LSD1 KO ESCs using the previously described DOX-inducible expression system^37^. Although the LSD1 level upon DOX induction in LSD1 recue (LSD1*Res*) cells was lower than that in WT ESCs (Supplementary Fig. 7a), LSD1 reintroduction suppressed genes upregulated by LSD1 deletion (Supplementary Fig. 7b–d). ChIP-Rx analysis indicated that LSD1 binds to its target regions upon DOX induction (Supplementary Fig. 7e–g). Importantly, LSD1 reintroduction caused a decrease of P300 occupancy at C1 and C2 clusters of LSD1 targeted active enhancers (Supplementary Fig. 7e, h), corroborating our results that LSD1 deletion caused the gain in P300 levels at these two clusters (Supplementary Fig. 6c). The decrease in P300 occupancy upon LSD1 re-introduction was correlated with the significant downregulation of genes near C1 and C2 clusters (Supplementary Fig. 7i), suggesting that LSD1 suppresses these genes through impeding the recruitment of P300 to LSD1 target enhancers. We noted that there are 81,645 LSD1 peaks in LSD1*Res* cells upon DOX induction, a number markedly higher than the 3182 LSD1 peaks in DKO + LSD1 cells after DOX induction. Moreover, 91% of LSD1 peaks in DKO + LSD1 cells upon DOX induction overlapped with that in LSD1*Res* cells (Supplementary Fig. 7j). HA ChIP-seq also showed a marked difference of peak numbers in LSD1*Res* and DKO + LSD1 cells upon DOX induction (Supplementary Fig. 7j). In addition, the increase of LSD1 ChIP-seq signals in LSD1*Res* cells upon DOX induction was significantly higher than that in DKO + LSD1 cells (Supplementary Fig. 7k), further indicating that the chromatin recruitment of LSD1 regulated by RCOR1/2 is critical for gene repression.

To examine the role of the H3K27ac gain at enhancers, we inhibited P300/CBP with the potent and specific inhibitor A485 in LSD1 KO cells^46^. Inhibition of P300/CBP led to a reduction of H3K27ac level globally and at LSD1 targets (Fig. 5e, f, Supplementary Fig. 8a). Furthermore, expression levels of ~22% LSD1-repressed genes were significantly downregulated upon P300/CBP inhibition in LSD1 KO cells (Figs. 1c and 5g, h). GSEA analysis demonstrated that LSD1-repressed genes are significantly downregulated by P300/CBP inhibition (Supplementary Fig. 8b), suggesting that the increase of H3K27ac at enhancers contributes to gene de-repression in LSD1 KO ESCs. We further investigated the role of P300/CBP’s acetyltransferase activity in the differentiation defect of LSD1 null ESCs. A485 treatment led to the downregulation of 32% de-repressed genes in LSD1 KO cells upon spontaneous differentiation (Supplementary Fig. 8c, d). Furthermore, ~26% of genes de-repressed by LSD1 deletion during EpiLC differentiation were suppressed by A485 treatment (Supplementary Fig. 8e, f). Surprisingly, A485 treatment of LSD1 KO cells during EB differentiation led to a 5-fold increase in EB sizes (Fig. 5i, j), indicating that the acetyltransferase activity of P300/CBP is partially responsible for the differentiation defect caused by LSD1 loss. GSEA analysis showed that genes de-repressed in LSD1 KO EBs are significantly suppressed by P300/CBP inhibition (Fig. 3c & Supplementary Fig. 8g). Specifically, ~22% genes suppressed by LSD1 during EB differentiation were significantly inactivated upon P300/CBP inhibition (Fig. 5k), further suggesting that P300 at LSD1 target enhancers contributes to differentiation failures caused by LSD1 deletion. Interestingly, neural related genes were highly enriched in these rescued genes in A485-treated LSD1 null EBs (Fig. 5l, m), suggesting that the upregulation of neural genes could cause differentiation defects of LSD1 KO ESCs. Altogether, our results demonstrated that the balance between LSD1 and P300/CBP is critical for the maintenance of the pluripotent transcriptome and the transcription reconfiguration required for cellular differentiation.

## Discussion

Although LSD1 is a well-established therapeutic target and has been heavily studied, the mechanisms underlying its role in gene regulation and cell fate transition remain unclear. Here we presented results indicating that LSD1 regulates gene expression and cellular differentiation through mechanisms independent from its demethylase activity in ESCs. We further demonstrated that LSD1 leads to enhancer decommissioning by impeding the recruitment of P300 to enhancers and that H3K27ac catalyzed by P300/CBP contributes to the differentiation failures caused by LSD1 loss (Fig. 5n).

We found that LSD1 catalytic inactivation results in a much milder perturbation to the ESC transcriptome than LSD1 deletion (Fig. 1). This is reminiscent of our findings that the catalytic function of H3K4 mono-methyltransferase MLL4 is largely dispensable for transcription regulation and exiting the naive pluripotency^6^. In recent years, catalytic-independent functions have been found in multiple epigenetic modifiers such as MLL3, MLL4, SET1A, SET1B, and DOT1L^6,10,47–49^, all of which harbor many functionally uncharacterized domains. In contrast, the structure of all LSD1 domains except for the N-terminal low complexity domain have been solved^32,33,39^. Three mutations in the catalytic amine oxidase-like (AOL) domain of LSD1 have been mapped in patients with a new genetic disorder that phenotypically resembles the Kabuki syndrome^20,21^. These mutations not only attenuate the catalytic activity of LSD1 but also impair the binding between LSD1 and transcription factors such as SNAIL1^50^, suggesting that the AOL domain harbors catalytic-independent functions. Indeed, the AOL domain is capable of binding to extra nucleosomal DNA besides binding histone tails and demethylating H3K4^33^. On the other hand, the TOWER domain interacts with CoREST complex while maintaining the accessibility to other proteins. The SWIRM domain may serve as an additional protein interacting hub for LSD1 to function at enhancers. Recently, a SWIRM domain missense mutation (R251Q) has been characterized in luminal breast cancer patients^51^. R251Q mutation leads to the increase in breast cancer cell migration and invasion potentially through impairing the capability of LSD1 to interact with other proteins. These lines of evidence point to the importance of studying proteins interacting with LSD1 besides the known interactors such as CoREST and NuRD complexes. A recent report has identified novel interactors of LSD1 through proximity labeling approaches^52^. Characterizing the functions of these interactions will facilitate the understanding of how LSD1 regulates gene expression. Identifying direct target enhancers and genes of LSD1 by rapid depletion is another important approach to decipher the mechanisms underlying the gene regulatory function of LSD1 since rapid protein degradation systems such as dTAG and AID^53–55^ circumvent potential secondary effects caused by shRNA guided depletion or constitutive deletion. Due to the importance of LSD1 in human diseases, developing inhibitors against its demethylase activity has attracted tremendous interest. Although more than seven inhibitors are going through different phases of clinical trials^27^, the mechanism of action of LSD1 inhibitors remains underexplored. Several LSD1 inhibitors have been shown to target the stability or protein interacting capability of LSD1 in addition to the demethylase activity^28,30,56,57^. Understanding how LSD1 inhibitors function using LSD1 KO and CI reagents in future studies is important for developing effective therapies to target diseases driven by LSD1 mutations or misregulation.

Although we did not observe global changes in H3K4me and H3K27ac levels in LSD1 KO or LSD1 CI cells, LSD1 deletion rather than catalytic inactivation leads to enhancer de-repression (Fig. 2). It is worth noting that both LSD1 deletion and inactivation cause increase of H3K4me1 and H3K4me2 levels at enhancers, suggesting that the removal of H3K4me1/2 by LSD1 at enhancers is insufficient for gene repression. This result is consistent with our findings that H3K4me1 at enhancers is largely dispensable for gene activation^6,10^; however, it raises an important question on what the function of LSD1’s demethylase activity is in gene regulation and cell fate transition. LSD1 catalytic inactivation leads to the misregulation of ~350 genes in ESCs, suggesting that the demethylase activity of LSD1 is required for fine-tuning gene expression. It is worth noting that the catalytic activity of LSD1 may play a more important role in different contexts such as in differentiated cells and diseases. It would be interesting to genetically examine the gene regulatory role of LSD1’s demethylase activity in other systems such as cancer cells that have upregulated LSD1 levels in future studies. We also noted that LSD1 deletion does not lead to the gain of H3K27ac at all LSD1-bound enhancers. H3K27ac level is mildly decreased in 37% of LSD1 peaks at active enhancers in LSD1 null ESCs. In addition to H3K4 demethylation, LSD1 can demethylate H3K9 and activate gene expression^58–60^. Whether the loss of LSD1 at specific enhancers causes the increase of H3K9me and leads to transcription inactivation is worth being examined in future studies. In addition, it is possible that the LSD1 targeting H3K4 and H3K9 are in different protein complexes. Identifying the context that affects LSD1’s choice to demethylate H3K4 or H3K9 is also very interesting.

Our results in four distinct differentiation strategies demonstrate that LSD1 catalytic inactivation has a much milder impact on cellular differentiation in comparison with LSD1 deletion (Fig. 3 and Supplementary Fig. 3). LSD1 protein is required for the proper expression of neural and cardiac genes during EB differentiation; however, its demethylase activity plays a less important role in regulating these genes. Our bulk RNA-seq and scRNA-seq results suggest a potential cell fate switch during lineage specification such that LSD1 loss leads to an increase in neuroectodermal cells and a decrease in meso-endodermal cells during differentiation. It would be interesting to study the function of neural gene upregulation during the differentiation of LSD1 depleted cells in future studies to test this hypothesis. It is noteworthy that ESC-like cells are more enriched in LSD1 KO compared with WT EBs, suggesting that LSD1 deletion impairs the capability of ESC to exit pluripotency during EB differentiation similar to spontaneous differentiation and EpiLC differentiation. Whether the increase of ESC-like cells contributes to the failure in meso-endoderm specification of LSD1 KO ESCs can be examined by depleting pluripotency factors in LSD1 KO cells during differentiation in future studies. We noted that LSD1 CI cells can contribute to cardiomyocytes although the cardiomyocyte network is less robust compared with WT ESCs. It would be important to study the catalytic function of LSD1 in cardiac differentiation in human stem cell models and in mice in the future to further delineate the mechanisms underlying human pathogenesis.

Our results suggest that CoREST components RCOR1/2 and LSD1 are functionally interdependent (Fig. 4). It has been demonstrated that LSD1 and RCOR1 are mutually stabilized^13,61^; however, we found that RCOR1 deletion does not alter the protein level of LSD1 or perturb the transcriptome in ESCs. It is possible that RCOR2, the major RCOR gene expressed in ESCs, compensates for RCOR1 in regulating the stability of LSD1. Indeed, RCOR2 deletion causes decreased LSD1 level and RCOR1/2 compound deletion leads to a further reduction of LSD1. Although RCOR2 level drops more than 2-fold in LSD1 KO cells, RCOR2 deletion does not recapitulate the transcription perturbation caused by LSD1 KO. On the other hand, the majority of misregulated genes in RCOR1/2 DKO ESCs and EBs overlap with that in their LSD1 KO counterparts, suggesting the redundancy between RCOR1 and RCOR2 as well as the interdependency between RCOR1/2 and LSD1 in regulating gene expression. Importantly, LSD1 deletion does not change the genome occupancy of RCOR2 and HDAC1/2, suggesting that LSD1 is not required for recruiting CoREST to chromatin. Based on the structure of CoREST complex, it is unlikely that RCOR1 and RCOR2 coexist in the same complex. We speculate that the RCOR2-containing CoREST and the RCOR1-containing CoREST simultaneously bind and suppress enhancers. Upon RCOR2 deletion, the residual amount of CoREST which mainly contains RCOR1 is sufficient to suppress most CoREST-targeted enhancers in the genome. Although RCOR1/2 deletion reduces LSD1 level in ESCs, overexpressing LSD1 does not significantly rescue gene misregulation in RCOR1/2 DKO cells. Interestingly, the restored LSD1 is capable of binding to chromatin albeit to much fewer binding sites and at a lower level compared with WT cells, indicating that RCOR1/2 regulate both LSD1 stability and the recruitment of LSD1 to chromatin. We noted that upon RCOR1/2 deletion and exogenous LSD1 introduction, LSD1 occupies about 4% of its binding sites in WT cells, suggesting CoREST independent mechanisms by which LSD1 is recruited to chromatin. LSD1 may bind to chromatin via its AOL domain or the interaction with other co-repressor complexes such as NuRD^33,62^. Future studies utilizing genetics and biochemistry approaches to dissect how LSD1 and other co-repressors coregulate enhancers are important to mechanistically understand enhancer decommissioning and gene regulation.

The elevation of enhancer H3K27ac levels after LSD1 deletion may be due to the loss of HDACs at these enhancers; however, our data suggest a model in which LSD1 impedes P300 from binding to its target enhancers (Fig. 5 and Supplementary Figs. 6, 7). Upon LSD1 loss, P300 is able to acetylate and activate its target enhancers, which leads to enhancer de-repression and failures in differentiation. It is noteworthy that the change in P300 and H3K27ac genome occupancy may not be a direct consequence of LSD1 deletion due to the potential secondary effects that could be caused by the constitutive knockout strategy. P300 could be recruited by transcription factors such as the KLF family of proteins upon LSD1 loss based on our motif analysis. Indeed, KLFs have been shown to recruit P300 and activate transcription in differentiated cells^63,64^. Since LSD1 is capable of binding DNA via its AOL domain^33^, it is possible that LSD1 occupies KLF binding sites such that the loss of LSD1 leads to the binding of KLFs to LSD1-targeted enhancers, which recruits P300 to de-repress LSD1 target genes. Besides transcription factors, the recruitment of P300 to enhancers could be regulated by epigenetic factors such as UTX, MLL4, and SWI/SNF^65–67^. Therefore, it would be interesting to search for epigenetic factors that can regulate the recruitment of P300 upon LSD1 loss. The regulation of P300 by LSD1 may not be limited to the chromatin recruitment of P300. There remains a possibility that LSD1 could dampen the acetyltransferase activity of P300/CBP at enhancers. LSD1 is known to interact with SirT1^68^, which has been shown to deacetylate P300 and reduce its activity^69^. It would be important to elucidate the functional relationship between LSD1 and P300/CBP via rapid protein degradation systems in the future. It is also noteworthy that our results do not exclude the potential role of HDACs in gene de-repression upon LSD1 loss. Although the genome occupancy of RCOR2 and HDACs is comparable in LSD1 KO and WT cells, it is possible that the activity of HDACs is diminished in LSD1 KO ESCs. Moreover, other HDACs besides HDAC1/2, which may play a role in deacetylating nucleosomes at enhancers, could be impaired by LSD1 loss. We also noted that the changes in P300 levels at LSD1 binding sites upon LSD1 loss do not always correlate with H3K27ac changes. The H3K27ac increase at enhancers in LSD1 KO ESCs may be induced by additional histone acetyltransferases (HATs). A comprehensive survey of histone acetylation levels by mass spectrometry and individually targeting HDACs and HATs in LSD1 null cells may provide more insights into how LSD1 regulates enhancers. Nonetheless, these studies need to be carefully designed as HDACs and HATs are associated with many different protein complexes. Targeting them may lead to confounding results due to the concurrent alteration of multiple pathways.

Our results indicate that the acetyltransferase activity of P300/CBP contributes to gene misregulation and differentiation defects of LSD1 null ESCs (Fig. 5 and Supplementary Fig. 8). Intriguingly, P300/CBP inhibition rescues the expression of many de-repressed genes by LSD1 deletion in both ESCs and EBs, suggesting that H3K27ac deposited by P300/CBP contributes to gene de-repression caused by LSD1 loss. It is noteworthy that P300/CBP have many non-histone substrates^70^. Future studies directly targeting H3K27ac and enhancer histone acetylation in LSD1 null mutants would be important to elucidate how histone acetylation contributes to enhancer-decommissioning caused by LSD1 loss. Interestingly, P300/CBP inhibition does not suppress all genes de-repressed by LSD1. We noticed that P300/CBP inhibition does not lead to the reduction of H3K27ac the same fashion globally. There are H3K27ac enriched regions less sensitive to P300/CBP inhibition, which could cause the resistance to transcription inactivation of genes regulated by these regions upon A485 treatment. It is also possible that other histone acetylation plays redundant roles to H3K27ac in de-repressing LSD1 target genes upon LSD1 loss. Whether H3K27me3 plays a role in suppressing genes sensitive to P300/CBP inhibition is also an interesting question. Understanding the crosstalk of different epigenetic pathways upon LSD1 depletion would be an important future study to understand mechanisms by which LSD1 catalytic-independently regulates enhancers and gene expression.

In summary, our study unveils a novel demethylase-independent role of LSD1 in regulating gene expression and cell fate transition of ESCs. Importantly, we reveal that the antagonism between LSD1 and P300 at enhancers is critical for the regulation of gene expression and differentiation. Based on our findings, it is critical to target LSD1 protein stability rather than the catalytic activity to design therapies against diseases driven by LSD1 overexpression. On the other hand, P300/CBP inhibition may serve as a novel approach for treating diseases caused by LSD1 loss-of-function.

## Methods

### Antibodies

The following primary antibodies were used in this study: anti-LSD1 (Abcam ab17721), anti-H3K4me1 (Cell Signaling Technology 5326), anti-H3K4me2 (Cell Signaling Technology 9725), Anti-H3K4me3 (Cell Signaling Technology 9727), anti-H3K27ac (Cell Signaling Technology 8173), anti-Tubulin (Developmental Studies Hybridoma Bank E7), anti-RCOR1 (Proteintech 27686-1-AP), anti-RCOR2 (Proteintech 23969-1-AP), anti-HDAC1 (Cell Signaling Technology 34589), anti-HDAC2 (Abcam ab7029), anti-H3 (Abcam ab1791), anti-P300 (Santa Cruz SC-48343X), anti-cTnT (Santa Cruz SC-20025), and anti-HA (Sigma H3663). The secondary antibodies used here were: donkey anti-rabbit IgG HRP (Sigma NA934V), sheep anti-mouse IgG HRP (Sigma NA931V), goat anti-mouse IgG-Alexa Fluor 488 (Life Technologies A11029), and goat anti-rabbit IgG-Alexa Fluor 594 (Life Technologies A32740).

### ESC culture and CRISPR/Cas9-guided gene editing

V6.5 ESCs^71^ were grown in N2B27 based serum free medium containing MEK inhibitor PD0325901 and GSK inhibitor CHIR99021 (2i), and LIF (Sigma) as previously described^6^. For doxycycline (DOX) induction, DKO + LSD1 or LSD1*Res* cells were treated with 2 µg/ml DOX for 48 h followed by downstream analyses. Alkaline Phosphatase (AP) staining was performed using the Alkaline Phosphatase Substrate Kit (Vector Laboratories) following manufacturer’s instructions. 60 percent confluent ESCs were subject to AP staining. For CRISPR guided gene knockout, desired guide RNAs (gRNAs) flanking the region of interest were synthesized from IDT, cloned, and transfected into cells using Amaxa Nucleofector II (Lonza) as previously described^72^. Cell clones were individually picked ten days after transfection and genotypes were determined using PCR. For knock-ins, the asymmetric single-stranded donor oligonucleotides^73^ and plasmids containing the desired gRNA were co-transfected into cells as described previously^47^. gRNA sequences and donor oligo sequences were as follows: LSD1 KO, TTGAAGAAGTGTTATGCGCC (left), AACAAACAAACTAACGCAGG (right); LSD1 K661 mutations, GGGATTTGGCAACCTTAACA (gRNA), CAGGCAAACATGGCTACATGCAGAGCCCATTCTTCACTGATGCTACCGGACACCAAATACTAAGCGACAGGAGAGAGGAAAGCAAAGCACCGCGTTAAGGTTGCCAAATCCCATCCTTTGGACTGCAGATGT (oligo donor for K661A), CAGGCAAACATGGCTACATGCAGAGCCCATTCTTCACTGATGCTACCGGACACCAAATACTAAGCGACAGGAGAGAGGAAAGCAAAGCACCTGGTTAAGGTTGCCAAATCCCATCCTTTGGACTGCAGATGT (oligo donor for K661Q); LSD1 A539E mutation, GGTAGAGAGAGGTGTGGCGT (gRNA), CCTGTACTCCCTGATTTTTTTTCAGTGATGTATACCTCTCATCAAGAGACAGACAAATACTTGACTGGCATTTTGCAAATCTTGAATTTGAGAACGCCACACCTCTCTCTACCCTCTCTCTTAAACATTGGG (oligo donor for A539E); RCOR1 KO, GTGTTTCATATTGCCGCCAG (left), TCTGGGAAGTCGTGCCAACA (right); RCOR2 KO, GGGGGTCGCAGTGAGCGTTA (left), GGATCTCTCTGGCAGCACTA (right).

### ESC differentiation

Spontaneous differentiation was performed by culturing naive ESCs for two passages (4 days) in Dulbecco’s modified Eagle’s medium (DMEM, Corning) supplemented with 15% FBS (Sigma), 1×penicillin-streptomycin (Life Technologies), 1×GlutaMAX (Life Technologies), 1×minimum essential medium nonessential amino acids (NEAA, Life Technologies), and 1×β-mercaptoethanol (Life Technologies). EpiLC differentiation was performed following the previously published protocol^37^. Briefly, naive ESCs were seeded on 6-well tissue culture dishes pre-treated with fibronectin (Millipore) and cultured 2 days with EpiLC media containing 1:1 mixed DMEM/F-12 (Life Technologies) and Neurobasal (Life Technologies) base media, 1×N2 (Life Technologies), 1×B27 (Life Technologies), 1×penicillin-streptomycin (Life Technologies), 1×GlutaMAX (Life Technologies), 1×minimum essential medium nonessential amino acids (Life Technologies), 1×β-mercaptoethanol (Life Technologies), 1% KOSR (Life Technologies), 20 ng/ml Activin A (R&D Systems), and 12 ng/ml FGF2 (R&D Systems).

Embryoid body (EB) differentiation was performed using the hanging-drop method with adaptation as previously described^48^. Briefly, 6 × 10^4^ cells/ml naive ESCs in EB differentiation medium were loaded on the lids of 15-cm petri dishes (Fisher) as 28 µL drops and cultured for 6 days. The components of EB differentiation medium are the same as the spontaneous differentiation medium described above. Size of EBs were quantified using Image J v1.53 (https://imagej.nih.gov/ij/).

High-efficiency cardiomyocyte differentiation was performed as previously described^38,74^. Briefly, naive ESCs were seeded onto petri dishes in serum-free differentiation (SFD) medium to form embryoid bodies. After 48 h, EBs were dissociated and reseeded onto petri dishes in SFD medium supplemented with 5 ng/ml VEGF, 0.25 ng/ml BMP4, and 5 ng/ml Activin A (all three cytokines from R&D Systems). After 48 h, mesodermal bodies were harvested, dissociated, and 1.25 × 10^5^–2.5 × 10^5^ cells were seeded on each chamber of the 0.1% gelatin precoated Nunc Lab-TeK chamber slide (Life Technologies) in cardiomyocyte differentiation medium (1×StemPro-34 medium supplemented with 2 mM L-glutamine (Life Technologies), 0.45 mM ascorbic acid, 5 ng/mL VEGF, 10 ng/mL FGF2, and 50 ng/mL FGF10). Media were changed every 24 h.

### Immunoprecipitation (IP)

Nuclear extract (NE) was prepared following previously published protocols^75^. In brief, nuclei were extracted under the low salt condition. NE was extracted under the high salt condition and treated with Benzonase (Sigma) to digest nucleic acid. For each IP, 5 μg antibodies were incubated with 1 mg NE overnight at 4 °C and incubated with Dynabeads Protein G (Life Technologies) for 2 h. Beads were washed and eluted in SDS loading buffer for Western blotting analysis.

### Immunofluorescence

Immunostaining was performed as previously described^47^. Briefly, cells were fixed with 4% paraformaldehyde, permeabilized with 0.2% TritonX-100, and blocked with 10% fetal bovine serum (FBS) in PBS. Cells were then stained with primary antibodies, stained with fluorescent secondary antibodies and DAPI dye in the dark, and washed again with 0.2% TritonX-100 in PBS. Coverslips were mounted using ProLong Gold antifade reagent (Life Technologies) and sealed with nail polish. Images were collected using an Olympus fluorescence microscope (model BX43F) with the 89-North PhotoFluor LM-75 light source.

### RNA-seq

All RNA-seq experiments were performed with at least two biological replicates from two independent cell clones. RNA was extracted and purified with Trizol reagent (Life Technologies) following manufacturer’s instructions. RNA was further treated with DnaseI (Sigma) and then purified with RNeasy mini kit (Qiagen). NEBNext rRNA Depletion Kit (New England BioLabs) and NEBNext Ultra II Directional RNA Kit (New England BioLabs) were used to deplete ribosomal RNA and prepare RNA-seq libraries, respectively. Libraries were pooled and sequenced on the HiSeq platform (Illumina) with a read length configuration of 150 bp on each end.

### Single cell RNA-seq (scRNA-seq)

scRNA-seq was performed on two biological replicates of day 6 EBs. EBs were dissociated by TrypLE (Life Technologies) and dissociated cells were resuspended in PBS + 0.04%BSA. The quantity and quality of the cells were accessed by Acridine Orange and Propidium iodide dye on a Cellometer Auto 2000 (Nexcelom). 16,000 cells were loaded on a 10x Chromium Controller based on 10x Chromium Single Cell 3’ Library manual (10x Genomics). After cell partitioning and GEM generation, reverse transcription was performed, and cDNA was pooled and cleaned up by beads. cDNA was further amplified and cleaned up by SPRI beads (Beckman). The quality of cDNA was assessed by High Sensitivity D5000 Tapestation (Agilent Technologies) and quantified by Qubit 2.0 DNA HS assay (Thermo Fisher). 3’ Gene expression library prep was carried out according to the 10x Chromium Next GEM Single Cell 3’ v3.1 manual. Equimolar pooling of libraries was performed based on QC values and sequenced on the NovaSeq platform (Illumina) with a read length configuration of 150 bp on each end.

### ChIP with reference exogenous genome (ChIP-Rx)

ChIP-Rx was performed as previously described^76^ with at least two biological replicates for each experiment presented in this study. In brief, ESCs were fixed with 1% formaldehyde (Thermo Fisher) and sheared with E220 focused ultrasonicator (Covaris), respectively. Sheared chromatins were mixed with 20% of lysate from HEK293T cells processed identically as spike-in for normalization. Mixed chromatin were incubated at 4 °C overnight with antibodies. Protein A/G beads (Santa Cruz Biotechnology) were then incubated with the chromatin and antibody mixture for 2 h at 4 °C. Beads were washed, reverse-crosslinked, and DNA were purified with Qiaquick PCR purification kit (Qiagen). KAPA HyperPrep Kit (Roche) was used to prepare ChIP-Rx libraries. Libraries were pooled and sequenced on the HiSeq platform (Illumina) with a read length configuration of 150 bp on each end.

### ChIP-Rx data analysis

ChIP-Rx mapping and peak calling were performed as previously published^77^. Raw reads were processed with Trim Galore v0.6.6 (https://www.bioinformatics.babraham.ac.uk/projects/trim_galore/) to remove adaptors and low-quality reads with the parameter “-q 25” and then aligned to the mouse mm9 and human hg19 genome assemblies using Bowtie v2.4.4 with default parameters^78^. All unmapped reads, low mapping quality reads (MAPQ < 30) and PCR duplicates were removed using SAMtools v1.12^79^ and Picard v2.25.5 (https://broadinstitute.github.io/picard/). The number of spike-in hg19 reads was counted with SAMtools v1.12 and normalization factor alpha = 1e6/hg19_count was calculated. Normalized bigwig was generated with bamCoverage function from deepTools v3.5.1 using scale factors calculated above and reads mapped to the ENCODE blacklist regions were removed using BEDTools v2.30.0^80–82^. Peaks were called using MACS2 v2.2.7.1 with option ‘nomodel’ and peak annotation was performed with R package ChIPseeker v1.28.3^83,84^. K-means clustering was performed and nearest-gene log changes in gene expression in the heat map of clustered peaks were generated using deepTools v3.5.1^82^. Overlapping and unique peaks were generated using findOverlaps function from R package GenomicRanges v1.46.0^85^. For occupancy boxplot representation at poised, intermediate, and active enhancers, normalized readcounts overlapping each region was calculated with getCountsByRegions function from R package BRGenomics v1.10.0 (https://mdeber.github.io) and log-transformed after adding a pseudo-count of 1. For occupancy boxplot representation from the clustered heatmaps, the matrix generated from deepTools v3.5.1^82^ was used to calculate the average coverage under each genome region.

### RNA-seq data analysis

Raw reads were trimmed as described in ChIP-Rx and then aligned to the mm9 genome assembly using STAR v2.7.9a with parameter “--outSAMtype BAM SortedByCoordinate --twopassMode Basic --outFilterMismatchNmax 2 --outSJfilterReads Unique”^86^. PCR duplicates were then filtered using Picard Tools v2.25.5 (https://broadinstitute.github.io/picard/). Reads were normalized to total read counts per million (cpm) and visualized as bigwig-formatted coverage tracks using deepTools v3.5.1^82^. Gene expression quantification was performed with featureCounts v2.0.2 with option ‘-s 2’^87^. Differential expression analysis was performed using R package DESeq2 v1.32.0^88^. Significant differentially expressed genes were filtered out with Benjamini-Hochberg-adjusted *p* values less than 0.01 and log fold change larger than |1|. Customed R scripts were used to generate heat maps and correlation plots. Boxplots and heat maps including hierarchical clustering were generated by ggboxplot function from package ggpubr v0.6.0 (https://rpkgs.datanovia.com/ggpubr/) and R package pheatmap v1.0.12 (https://cran.r-project.org/web/packages/pheatmap/index.html), respectively. GSEA analysis was performed by R package clusterProfiler v4.6.2^89^. Gene ontology analyses were performed using Metascape v3.5^90^.

### scRNA-seq data analysis

scRNA-seq reads were aligned and counted by Cell Ranger v7.1.0^91^ with default parameters. Filtering, clustering, and assigning cell type identity to clusters were performed using R package Seurat v4.3.0^92^. Briefly, genes expressed in less than 10 cells and cells in which total UMI < 500 or total expressed genes <500 were removed. Gene counts were normalized by NormalizeData function. 2000 highly variable genes were selected in each sample based on a variance stabilizing transformation performed by FindVariableFeatures function. Next, expression matrices were scaled and centered followed by principal component analysis (PCA) for dimensional reduction. PC1 to PC30 were used to construct nearest neighbor graphs in the PCA space (FindNeighbors function) followed by Louvain clustering to identify clusters (resolution = 0.4, FindClusters function). Then, cell clusters were assigned to ESCs, endoderm cells, mesoderm cells and ectoderm cells by their canonical gene markers. ESCs were marked with *Klf2*, *Zfp42*, *Pou5f1*, and *Sox2*. Endoderm cells were marked with *Cldn6* and *Sox17*. Mesoderm cells were marked with *Hand2*, *Hand1*, *Tnnt2*, and *Mef2c*. Ectoderm cells were marked with *Pou3f1* and *Nes*.

### Reporting summary

Further information on research design is available in the Nature Portfolio Reporting Summary linked to this article.

### Supplementary information

Supplementary Information

Reporting Summary

### Source data

Source Data

## Data Availability

The raw and processed high-throughput sequencing datasets including ChIP-Rx, RNA-seq, and scRNA-seq generated in this study have been deposited to the Gene Expression Omnibus (GEO) database under the accession number GSE232255 and can be downloaded from the link below. Information about the mm9 genome assembly can be found at https://www.ncbi.nlm.nih.gov/assembly/GCF_000001635.18/. Details on oligonucleotide sequences, antibodies, and additional reagents are listed in the “Methods” section. All remaining data associated with this study are available within the Article and Supplementary Data. Source data are provided with this paper.
