# Supplementary material for: Demethylase-independent roles of LSD1 in regulating enhancers and cell fate transition
Source: Nat Commun. 2023 Aug 22;14:4944. doi: 10.1038/s41467-023-40606-1 (PMC10444793; doi:10.1038/s41467-023-40606-1)
Supplement: Supplementary file 1 — Supplementary Information [file 41467_2023_40606_MOESM1_ESM.pdf]

Supplementary information for

Demethylase-independent roles of LSD1 in regulating enhancers and cell fate transition

Cheng Zeng, Jiwei Chen, Emmalee W. Cooke, Arijita Subuddhi, Eliana T. Roodman, Fei Xavier Chen, and Kaixiang Cao

Correspondence should be sent to the following address:

Kaixiang Cao, PhD

Assistant Professor

Department of Biochemistry, Case Western Reserve University

10900 Euclid Avenue, Cleveland, OH 44106, USA

Office: (216) 368-1218

Email: kxc725@case.edu

Supplementary information includes 8 Supplementary Figures.

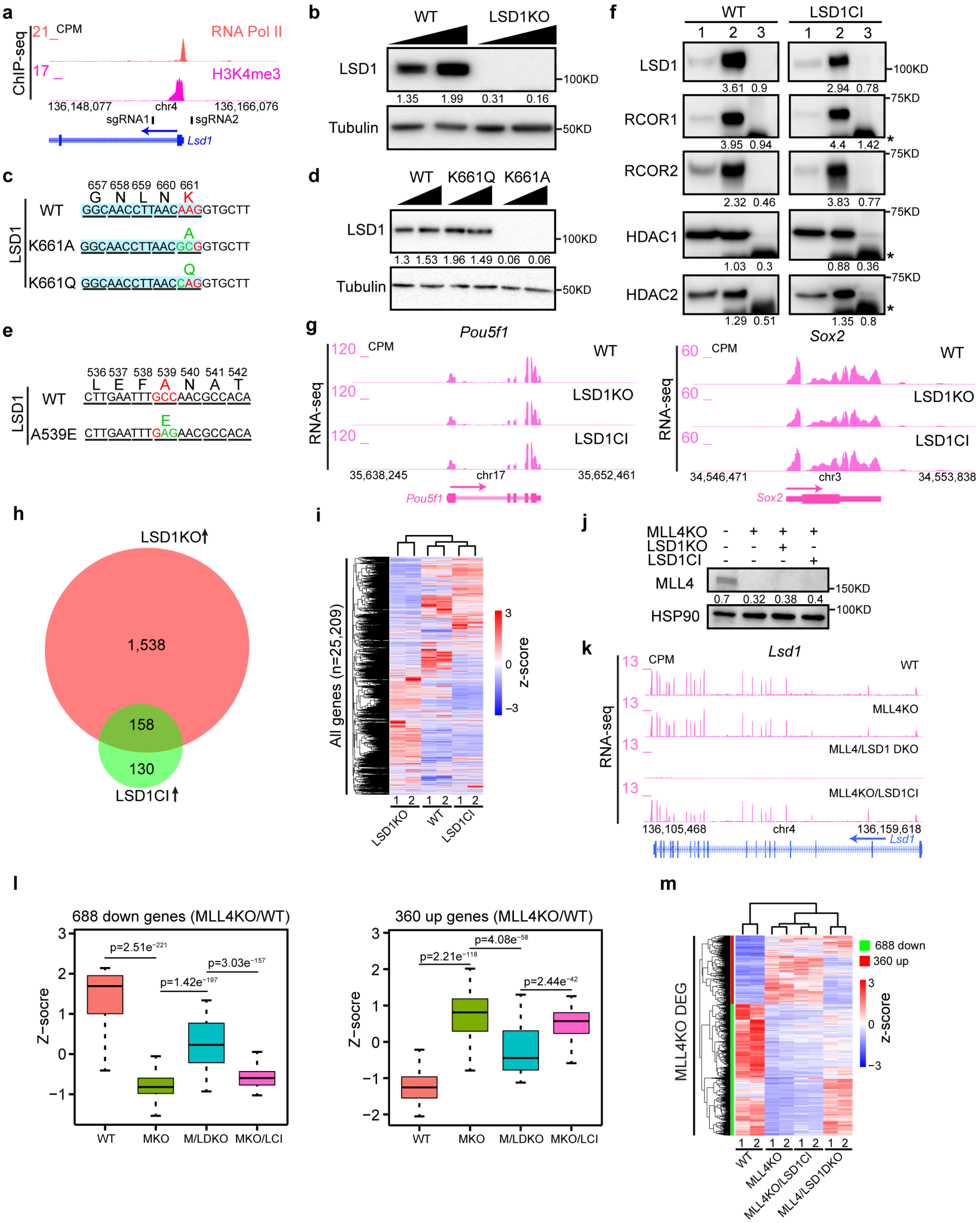
a
ChIP-seq
21_ CPM
RNA Pol II
17 _
H3K4me3
136,148,077
chr4
136,166,076
sgRNA1
sgRNA2
Lsd1
b
WT
LSD1KO
LSD1
100KD
1.35 1.99 0.31 0.16
Tubulin
50KD
c
657 658 659 660 661
G N L N K
WT GGCAACCTTAACAAGGTGCTT
LSD1
A
K661A GGCAACCTTAACGCGGTGCTT
Q
K661Q GGCAACCTTAACCAGGTGCTT
d
WT K661Q K661A
LSD1
100KD
1.3 1.53 1.96 1.49 0.06 0.06
Tubulin
50KD
e
536 537 538 539 540 541 542
L E F A N A T
WT CTTGAATTTGCCAACGCCACA
LSD1
E
A539E CTTGAATTTGAGAACGCCACA
f
WT
LSD1CI
1 2 3 1 2 3
LSD1
100KD
3.61 0.9 2.94 0.78
RCOR1
75KD
3.95 0.94 4.4 1.42
RCOR2
75KD
2.32 0.46 3.83 0.77
HDAC1
75KD
1.03 0.3 0.88 0.36
HDAC2
75KD
1.29 0.51 1.35 0.8
g
RNA-seq
Pou5f1
120 _ CPM
WT
120 _
LSD1KO
120 _
LSD1CI
35,638,245 chr17 35,652,461
Pou5f1
Sox2
60 _ CPM
WT
60 _
LSD1KO
60 _
LSD1CI
34,546,471 chr3 34,553,838
Sox2
h
LSD1KO↑
1,538
158
130
LSD1CI↑
i
All genes (n=25,209)
3
0
−3
z-score
1 2 1 2 1 2
LSD1KO WT LSD1CI
j
MLL4KO - + + +
LSD1KO - - + -
LSD1CI - - - +
MLL4
150KD
0.7 0.32 0.38 0.4
HSP90
100KD
k
RNA-seq
Lsd1
13 _ CPM
WT
13 _
MLL4KO
13 _
MLL4/LSD1 DKO
13 _
MLL4KO/LSD1CI
136,105,468 chr4 136,159,618
Lsd1
l
688 down genes (MLL4KO/WT)
Z-socre
p=2.51e−221
p=1.42e−197
p=3.03e−157
WT MKO M/LDKO MKO/LCI
360 up genes (MLL4KO/WT)
Z-socre
p=4.08e−58
p=2.21e−118
p=2.44e−42
WT MKO M/LDKO MKO/LCI
m
MLL4KO DEG
688 down
360 up
3
0
−3
z-score
1 2 1 2 1 2 1 2
WT MLL4KO MLL4KO/LSD1CI MLL4/LSD1DKO

Supplementary Figure 1. Generation and characterization of LSD1 KO and LSD1 CI ESCs.
(a) The genome location of sgRNAs used to delete LSD1.
(b) Western blotting showing the loss of LSD1 in LSD1 KO ESCs. Experiments were repeated three times independently with similar results observed. Source data are provided as a Source Data file.
(c) Sequences of the WT, LSD1 K661A, and K661Q mutant alleles. The exonic region is highlighted in blue.
(d) Western blotting indicating LSD1 levels in WT, K661Q, and K661A ESCs. Experiments were repeated three times independently with similar results observed. Source data are provided as a Source Data file.
(e) Sequences of the WT and LSD1 A539E alleles.
(f) Western blotting indicating WT and K661Q/A539E mutant LSD1 co-immunoprecipitate (co-IP) with CoREST components RCOR1, RCOR2, HDAC1, and HDAC2. Lane 1: 4% input; lane 2: LSD1 IP; lane 3: IgG IP. *: IgG heavy chain. LSD1 IP and IgG IP lanes were quantified by normalizing with the corresponding input lanes. Experiments were repeated three times independently with similar results observed. Source data are provided as a Source Data file.
(g) Genome browser view of RNA-seq signals at *Pou5f1* and *Sox2* genes in WT, LSD1 KO and LSD1 CI ESCs.
(h) Venn diagram indicating the overlap between genes upregulated in LSD1 KO and CI ESCs.
(i) Hierarchical clustering analysis of expression of all 25,209 genes comparing WT, LSD1 KO, and LSD1 CI ESCs. Numbers below the heatmap denote the 2 biological replicates of each genotype.
(j) Western blotting indicating the loss of MLL4 in MLL4KO, LSD1/MLL4 DKO, and LSD1 CI/MLL4 KO ESCs. Experiments were repeated three times independently with similar results observed. Source data are provided as a Source Data file.
(k) Genome browser view of RNA-seq signals at LSD1 gene in cells mentioned in (j).
(l) Box plots quantifying the levels of genes downregulated (left) and upregulated (right) by MLL4 deletion in labelled cells. MKO: MLL4KO, M/LDKO: MLL4/LSD1 DKO, MKO/LCI: MLL4KO/LSD1CI. n=2 biologically independent experiments. P-values were calculated using two-sided Wilcoxon signed-rank tests. Center line: median; top and bottom hinges of box: the third and first quantiles; whiskers: quartiles ± 1.5 × interquartile range.
(m) Hierarchical clustering analysis of expression levels of the differentially regulated genes in MLL4 KO cells comparing WT, MLL4 KO, MLL4/LSD1 DKO, and MLL4KO/LSD1CI ESCs. Numbers below the heatmap denote the 2 biological replicates of each genotype.

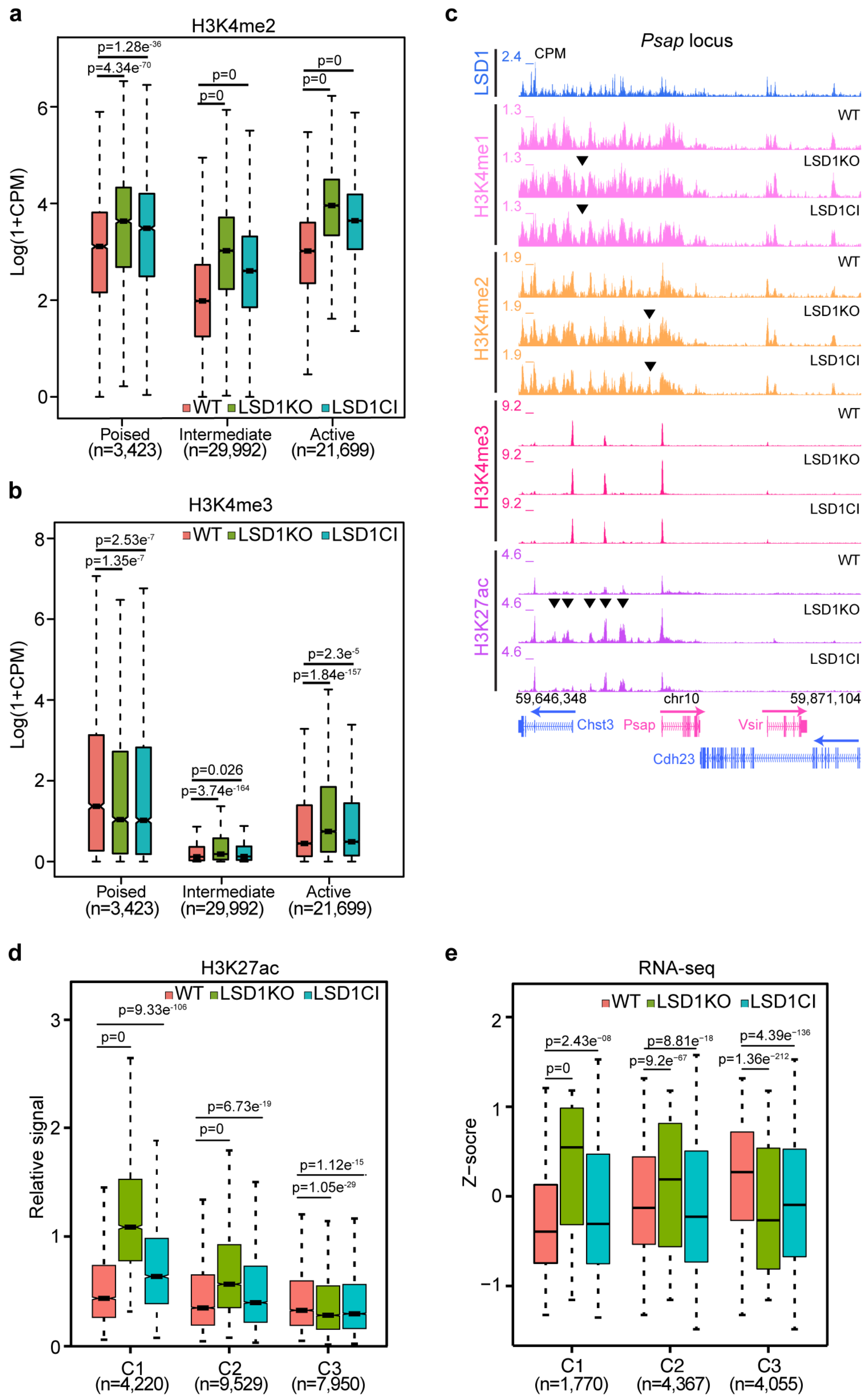
a
H3K4me2
Log(1+CPM)
p=1.28e-36
p=4.34e-70
p=0
p=0
p=0
p=0
WT LSD1KO LSD1CI
Poised (n=3,423)
Intermediate (n=29,992)
Active (n=21,699)
b
H3K4me3
Log(1+CPM)
p=2.53e-7
p=1.35e-7
p=0.026
p=3.74e-164
p=2.3e-5
p=1.84e-157
WT LSD1KO LSD1CI
Poised (n=3,423)
Intermediate (n=29,992)
Active (n=21,699)
c
Psap locus
CPM
LSD1
H3K4me1
H3K4me2
H3K4me3
H3K27ac
WT
LSD1KO
LSD1CI
59,646,348
chr10
59,871,104
Chst3
Psap
Vsir
Cdh23
d
H3K27ac
Relative signal
WT LSD1KO LSD1CI
p=9.33e-106
p=0
p=6.73e-19
p=0
p=1.12e-15
p=1.05e-29
C1 (n=4,220)
C2 (n=9,529)
C3 (n=7,950)
e
RNA-seq
Z−socre
WT LSD1KO LSD1CI
p=2.43e-08
p=0
p=8.81e-18
p=9.2e-67
p=4.39e-136
p=1.36e-212
C1 (n=1,770)
C2 (n=4,367)
C3 (n=4,055)

Supplementary Figure 2. The impact of LSD1 deletion and catalytic inactivation on enhancer histone modifications.

(a-b) Box plots of H3K4me2 (a) and H3K4me3 (b) levels at LSD1 bound enhancers in WT, LSD1 KO, and LSD1 CI cells. 3,423 poised, 29,992 intermediate, and 21,699 active enhancers were called based on H3K4me1, H3K27ac, H3K27me3, and LSD1 ChIP-Rx data. n=2 biologically independent experiments. P-values (p) from two-sided Wilcoxon signed-rank tests on log2(LSD1KO/WT) and log2(LSD1CI/WT) are denoted in each panel. Center line: median; top and bottom hinges of box: the third and first quantiles; whiskers: quartiles $\pm$ 1.5 $\times$ interquartile range.

(c) Genome browser view of H3K4me1/2/3 and H3K27ac ChIP-Rx signals at the *Psap* locus in WT and LSD1 KO, and LSD1 CI ESCs. Black arrows indicate changes between LSD1 mutant and WT cells.

(d) Box plots showing H3K27ac levels of WT, LSD1 KO, and LSD1 CI ESCs at the three clusters of LSD1 bound active enhancers in Fig. 2e. P-values were calculated using two-sided Wilcoxon signed-rank tests. Center line: median; top and bottom hinges of box: the third and first quantiles; whiskers: quartiles $\pm$ 1.5 $\times$ interquartile range.

(e) Box plots showing RNA-seq signals in WT, LSD1 KO, and LSD1 CI ESCs of nearest gene to the three clusters of LSD1 bound active enhancers in Fig. 2e. Z-scores were used to generate the heat map. P-values were calculated using two-sided Wilcoxon signed-rank tests. Center line: median; top and bottom hinges of box: the third and first quantiles; whiskers: quartiles $\pm$ 1.5 $\times$ interquartile range.

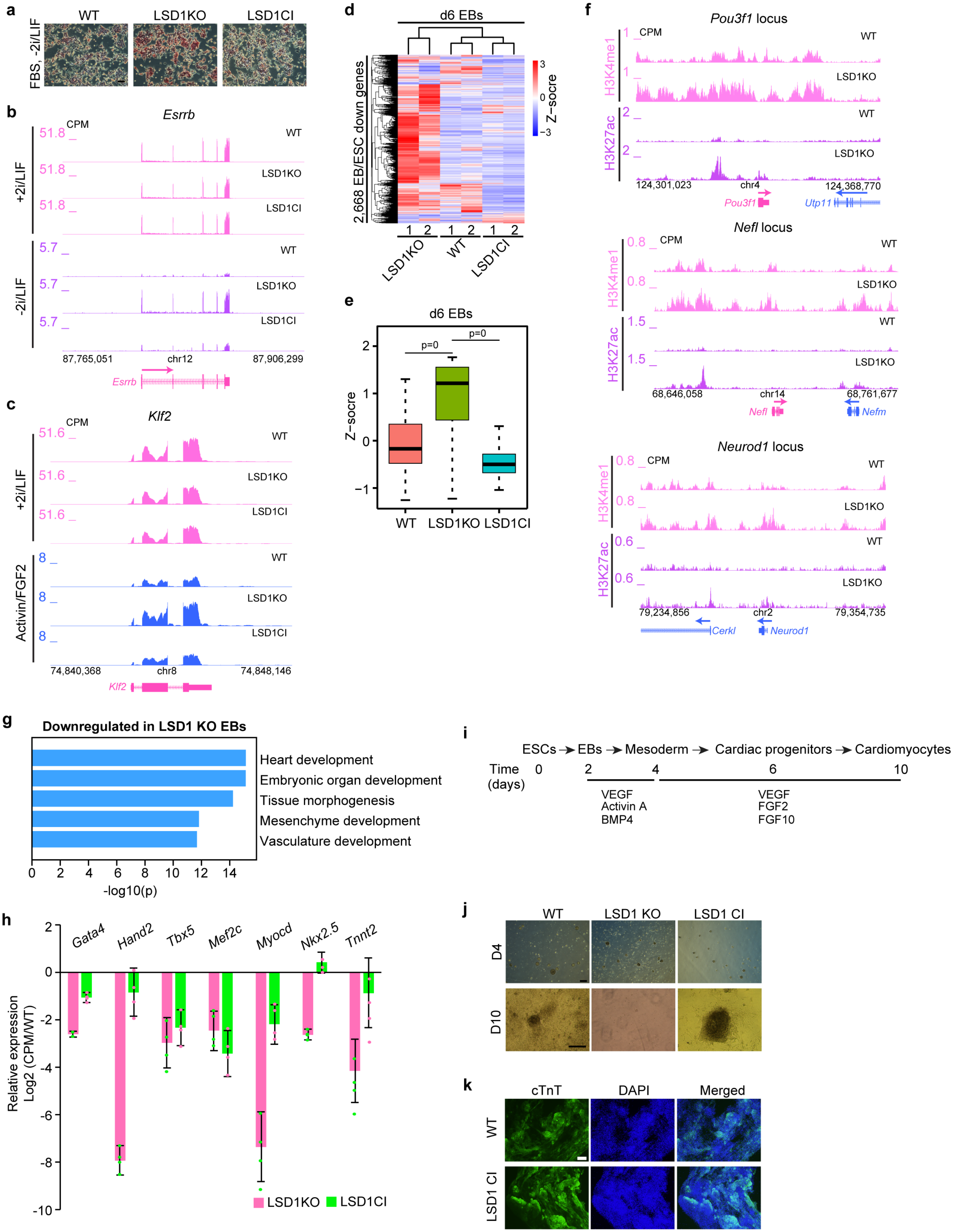

a
WT
LSD1KO
LSD1CI
FBS, -2i/LIF
b
Esrrb
CPM
+2i/LIF
-2i/LIF
51.8
5.7
87,765,051
chr12
87,906,299
c
Klf2
51.6
8
Activin/FGF2
74,840,368
chr8
74,848,146
d
d6 EBs
2,668 EB/ESC down genes
Z-socre
3
0
−3
1 2 1 2 1 2
e
d6 EBs
p=0
p=0
Z-socre
WT LSD1KO LSD1CI
f
Pou3f1 locus
H3K4me1
H3K27ac
124,301,023
chr4
124,368,770
Pou3f1
Utp11
Nefl locus
68,646,058
chr14
68,761,677
Nefl
Nefm
Neurod1 locus
79,234,856
chr2
79,354,735
Cerkl
Neurod1
g
Downregulated in LSD1 KO EBs
Heart development
Embryonic organ development
Tissue morphogenesis
Mesenchyme development
Vasculature development
-log10(p)
h
Gata4
Hand2
Tbx5
Mef2c
Myocd
Nkx2.5
Tnnt2
Relative expression
Log2 (CPM/WT)
LSD1KO
LSD1CI
i
ESCs → EBs → Mesoderm → Cardiac progenitors → Cardiomyocytes
Time (days) 0 2 4 6 10
VEGF
Activin A
BMP4
VEGF
FGF2
FGF10
j
WT
LSD1 KO
LSD1 CI
D4
D10
k
cTnT
DAPI
Merged
WT
LSD1 CI

Supplementary Figure 3. The impact of LSD1 deletion and inactivation on ESC differentiation.
(a) AP staining of WT, LSD1 KO, and LSD1 CI cells two passages after 2i/LIF withdrawal. Experiments were repeated three times independently with similar results observed. Scale bar: 100μm.
(b) Genome browser view of RNA-seq signals at *Esrrb* gene in WT and LSD1 mutants under naive state (top) and spontaneous differentiation (bottom).
(c) Genome browser view of RNA-seq signals at *Klf2* gene in WT and LSD1 mutants under naive (top) and primed (bottom) states.
(d-e) Hierarchical clustering (d) and box plot (e) analyses of the expression levels of 2,668 genes downregulated during EB differentiation in day 6 WT, LSD1 KO, and LSD1 CI EBs. P-values in (e) were calculated using two-sided Wilcoxon signed-rank tests. Center line: median; top and bottom hinges of box: the third and first quantiles; whiskers: quartiles ± 1.5 × interquartile range.
(f) Genome browser view of H3K4me1 and H3K27ac levels in WT and LSD1 KO EBs at neural marker gene loci.
(g) GO analysis of genes downregulated in LSD1 KO day 6 EBs vs. WT EBs.
(h) Fold change of RNA-seq signals (CPM) in LSD1 KO or LSD1 CI over WT EBs is shown for cardiac marker genes. n=2 biologically independent experiments. Data are presented as mean values ± SD. Source data are provided as a Source Data file.
(i) The cardiac differentiation strategy.
(j) Phase contrast images of day 4 (top) and day 10 (bottom) cardiac differentiation cultures of WT, LSD1 KO, and LSD1 CI cells. Experiments were repeated three times independently with similar results observed. Scale bar: 250μm.
(k) Immunostaining images of WT and LSD1 CI cardiac differentiation cultures at day 10. Cells were stained with anti-cTnT antibodies and DAPI. Experiments were repeated three times independently with similar results observed. Scale bar: 100μm.

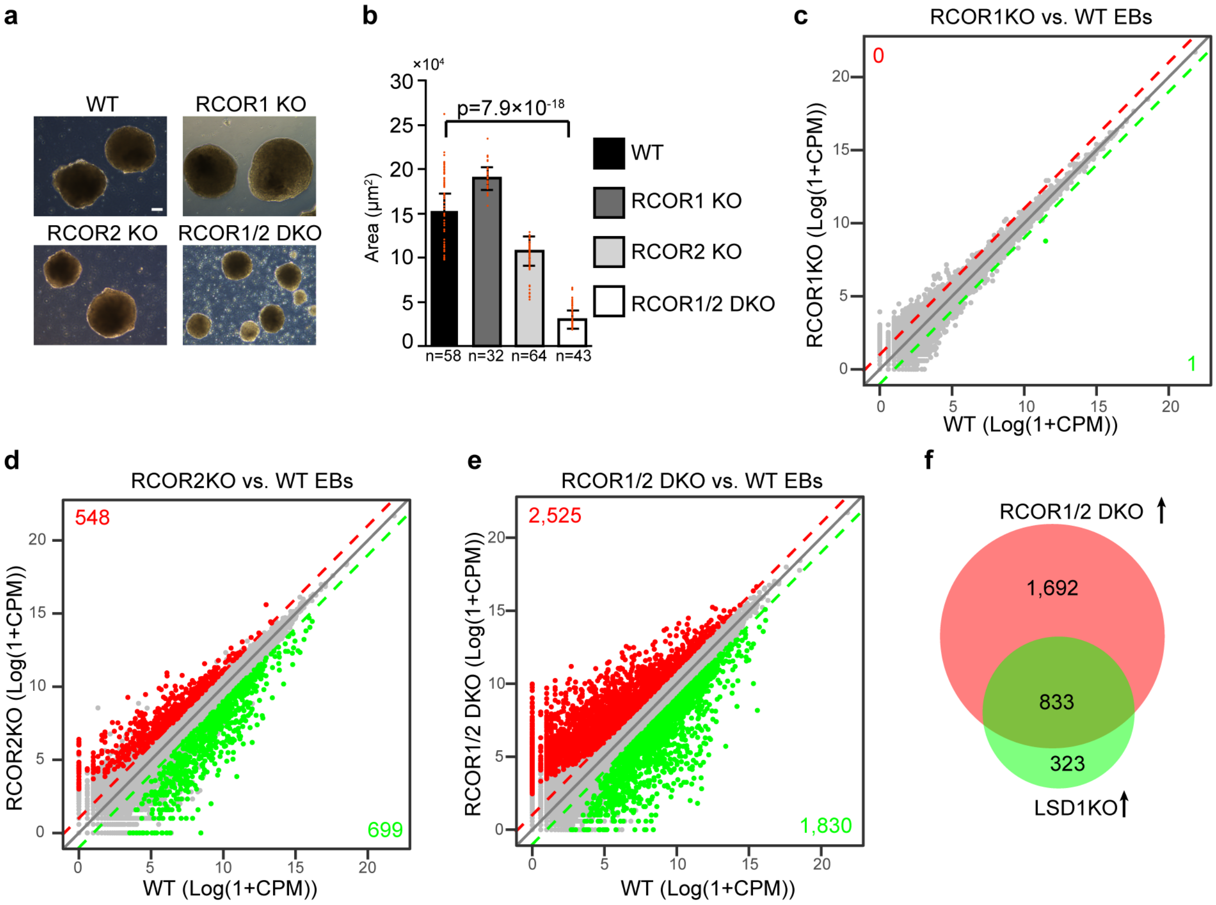
a
WT
RCOR1 KO
RCOR2 KO
RCOR1/2 DKO
b
×10⁴
p=7.9×10⁻¹⁸
Area (μm²)
n=58 n=32 n=64 n=43
WT
RCOR1 KO
RCOR2 KO
RCOR1/2 DKO
c
RCOR1KO vs. WT EBs
0
1
RCOR1KO (Log(1+CPM))
WT (Log(1+CPM))
d
RCOR2KO vs. WT EBs
548
699
RCOR2KO (Log(1+CPM))
WT (Log(1+CPM))
e
RCOR1/2 DKO vs. WT EBs
2,525
1,830
RCOR1/2 DKO (Log(1+CPM))
WT (Log(1+CPM))
f
RCOR1/2 DKO ↑
1,692
833
323
LSD1KO↑

Supplementary Figure 4. RCOR1/2 deletion impairs EB differentiation.
(a) Phase contrast images of day 6 EBs generated from WT, RCOR1 KO, RCOR2 KO, and RCOR1/2 DKO ESCs. Experiments were repeated three times independently with similar results observed. Scale bar: 100μm.
(b) Quantification of EB sizes in (a). Data are presented as mean values ± SD. n=3 biologically independent experiments. P-values were calculated using two-sided student's t-test. Source data are provided as a Source Data file.
(c-e) Correlation plots of RNA-seq data between RCOR1 KO (c), RCOR2 KO (d), and RCOR1/2 DKO (e) EBs and WT EBs. Statistical significance was determined by two-sided Wald test and p-values were corrected for multiple testing using the Benjamini-Hochberg method. Significantly up- and downregulated genes are labelled in red and green with numbers of genes noted, respectively.
(f) Venn diagram showing the overlap of upregulated genes between LSD1 KO and RCOR1/2 DKO EBs compared with WT EBs.

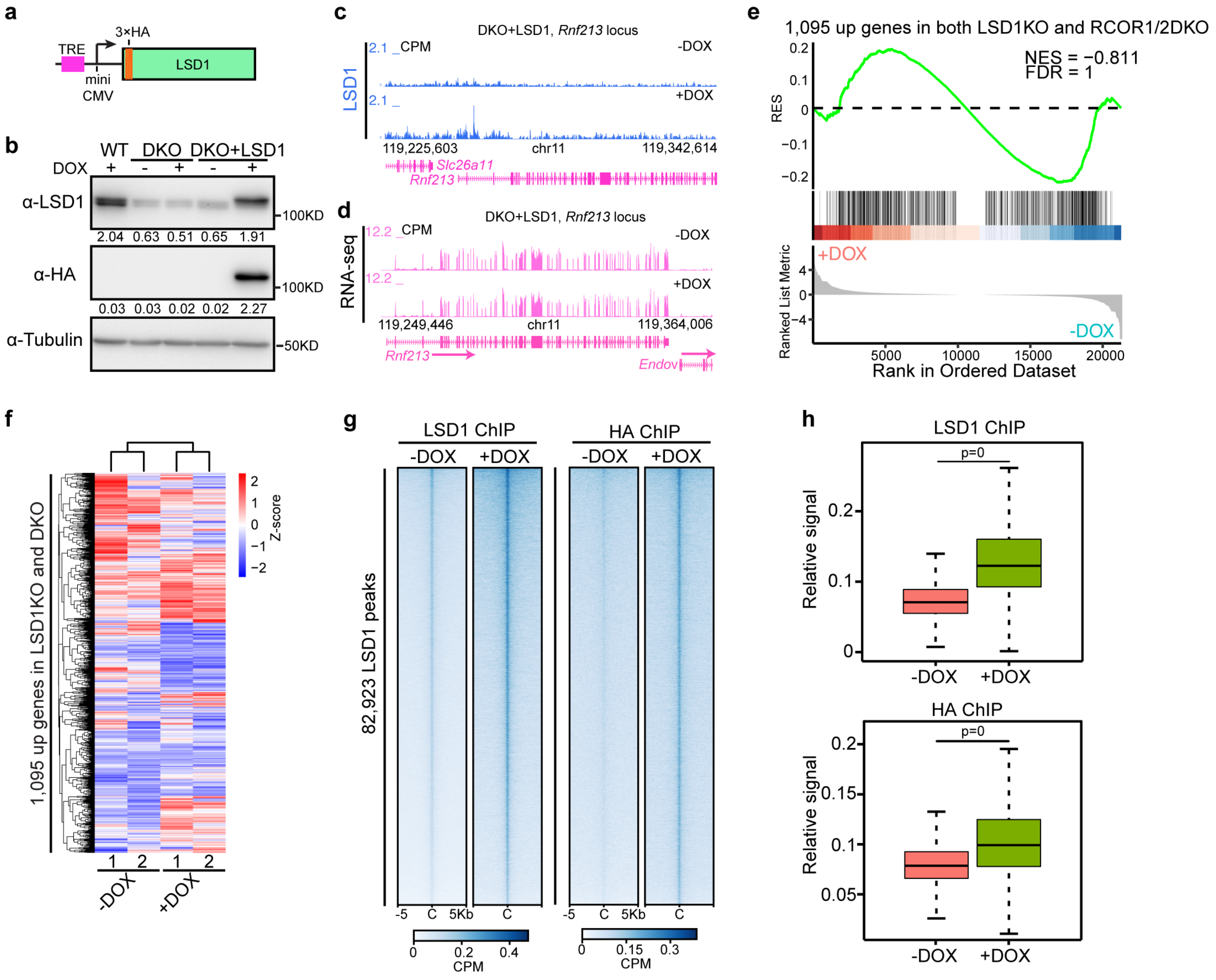
a
TRE
3×HA
mini CMV
LSD1
b
WT DKO DKO+LSD1
DOX
α-LSD1
2.04 0.63 0.51 0.65 1.91
α-HA
0.03 0.03 0.02 0.02 2.27
α-Tubulin
100KD
50KD
c
DKO+LSD1, Rnf213 locus
LSD1
CPM
-DOX
+DOX
119,225,603 chr11 119,342,614
Slc26a11
Rnf213
d
DKO+LSD1, Rnf213 locus
RNA-seq
119,249,446 chr11 119,364,006
Endov
e
1,095 up genes in both LSD1KO and RCOR1/2DKO
NES = −0.811
FDR = 1
RES
Ranked List Metric
Rank in Ordered Dataset
f
1,095 up genes in LSD1KO and DKO
Z-score
g
LSD1 ChIP
HA ChIP
82,923 LSD1 peaks
CPM
h
LSD1 ChIP
HA ChIP
p=0
Relative signal

Supplementary Figure 5. LSD1 restoration has no significant impact on rescuing de-repressed genes in CoREST null cells.
(a) Schematic representation of the doxycycline (DOX) inducible LSD1 overexpressing system. TRE: Tetracycline response element.
(b) Western blotting analysis of DKO+LSD1 cells with and without DOX induction. WT and RCOR1/2 DKO cell lysates served as controls. Levels of LSD1 and HA-LSD1 in each lane were quantified by normalizing with Tubulin signals of the corresponding lane. Normalized ratios were provided under each blot. Experiments were repeated three times independently with similar results observed. Source data are provided as a Source Data file.
(c) Genome browser view of LSD1 ChIP-Rx signals at the *Rnf213* locus in DKO+LSD1 cells with and without DOX induction.
(d) Genome browser view of RNA-seq signals of *Rnf213* gene in DKO+LSD1 cells with and without DOX induction.
(e) GSEA analysis of 1,095 genes upregulated by both LSD1 and RCOR1/2 loss in the right panel of Fig. 4f comparing DKO+LSD1 cells with and without DOX induction.
(f) Hierarchical analysis of 1,095 genes upregulated by both LSD1 and RCOR1/2 loss in DKO+LSD1 cells with and without DOX induction. Numbers below the heatmap denote the 2 biological replicates of each condition.
(g) Heatmap analysis showing LSD1 ChIP-Rx (left) and HA ChIP-seq (right) signals in DKO+LSD1 cells with and without DOX induction. Signals were centered on total LSD1 peaks and the profiles were sorted in a descending order of LSD1 occupancy in WT ESCs.
(h) Box plot analysis of LSD1 ChIP-Rx (top) and HA ChIP-seq (bottom) signals in DKO+LSD1 cells with and without DOX induction at 82,923 LSD1 peaks. n=2 biologically independent experiments. Center line: median; top and bottom hinges of box: the third and first quantiles; whiskers: quartiles $\pm 1.5 \times$ interquartile range. P-values were calculated with two-sided Wilcoxon signed-rank tests.

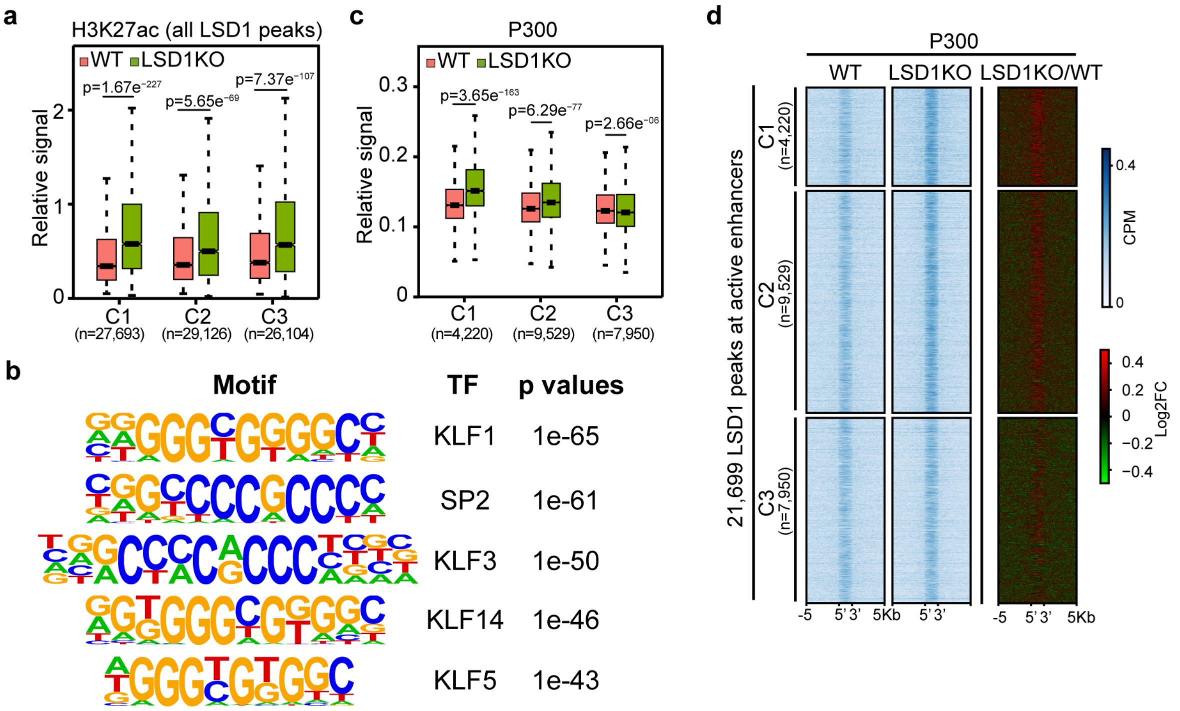
a
H3K27ac (all LSD1 peaks)
WT LSD1KO
p=1.67e-227
p=5.65e-69
p=7.37e-107
Relative signal
C1 (n=27,693)
C2 (n=29,126)
C3 (n=26,104)
b
Motif
TF
p values
KLF1 1e-65
SP2 1e-61
KLF3 1e-50
KLF14 1e-46
KLF5 1e-43
c
P300
WT LSD1KO
p=3.65e-163
p=6.29e-77
p=2.66e-06
Relative signal
C1 (n=4,220)
C2 (n=9,529)
C3 (n=7,950)
d
P300
WT
LSD1KO
LSD1KO/WT
21,699 LSD1 peaks at active enhancers
C1 (n=4,220)
C2 (n=9,529)
C3 (n=7,950)
CPM
Log2FC
-5 5' 3' 5Kb

Supplementary Figure 6. LSD1 deletion leads to increased P300 recruitment to enhancers.
(a) Box plot analysis of H3K27ac levels in WT and LSD1 KO ESCs at all 82,923 LSD1 peaks according to the three clusters in Fig. 5b. Center line: median; top and bottom hinges of box: the third and first quantiles; whiskers: quartiles ± 1.5 × interquartile range. P-values were calculated with two-sided Wilcoxon signed-rank tests.
(b) Top transcription factors motifs enriched in Cluster 1 of all 82,923 LSD1-enriched regions in Fig. 5b. Motif analysis was performed using Cluster 2 and 3 regions as controls. P-values were calculated using an one-tailed hypergeometric test.
(c) Box plot analysis of P300 levels in WT and LSD1 KO ESCs at 21,699 LSD1 enriched active enhancers according to the three clusters in Fig. 2e. Center line: median; top and bottom hinges of box: the third and first quantiles; whiskers: quartiles ± 1.5 × interquartile range. P-values were calculated with two-sided Wilcoxon signed-rank tests.
(d) Heatmap analysis indicating P300 occupancy (left) and log 2 fold change (right) at 21,699 LSD1 enriched active enhancers. Clusters are the same as in Fig. 2e.

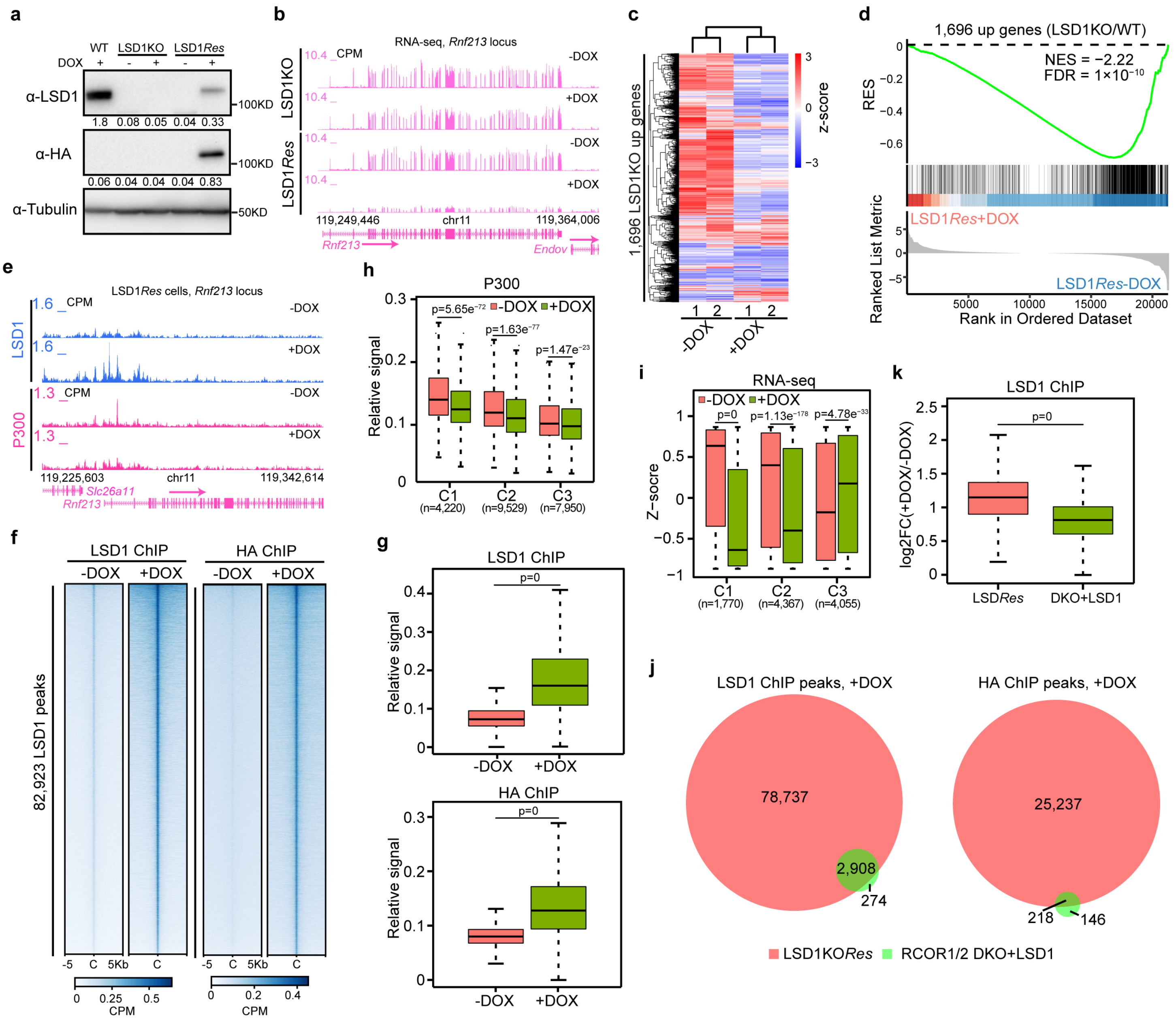
a
WT
LSD1KO
LSD1Res
DOX
α-LSD1
100KD
1.8 0.08 0.05 0.04 0.33
α-HA
100KD
0.06 0.04 0.04 0.04 0.83
α-Tubulin
50KD
b
RNA-seq, Rnf213 locus
LSD1KO
LSD1Res
CPM
-DOX
+DOX
119,249,446
chr11
119,364,006
Rnf213
Endov
c
1,696 LSD1KO up genes
z-score
-DOX
+DOX
d
1,696 up genes (LSD1KO/WT)
NES = −2.22
FDR = 1×10−10
RES
LSD1Res+DOX
LSD1Res-DOX
Ranked List Metric
Rank in Ordered Dataset
e
LSD1Res cells, Rnf213 locus
LSD1
P300
119,225,603
chr11
119,342,614
Slc26a11
Rnf213
h
P300
Relative signal
p=5.65e−72
p=1.63e−77
p=1.47e−23
C1 (n=4,220)
C2 (n=9,529)
C3 (n=7,950)
i
RNA-seq
Z-socre
p=0
p=1.13e−178
p=4.78e−33
C1 (n=1,770)
C2 (n=4,367)
C3 (n=4,055)
k
LSD1 ChIP
log2FC(+DOX/-DOX)
p=0
LSDRes
DKO+LSD1
f
LSD1 ChIP
HA ChIP
82,923 LSD1 peaks
5Kb
CPM
g
LSD1 ChIP
HA ChIP
Relative signal
p=0
j
LSD1 ChIP peaks, +DOX
HA ChIP peaks, +DOX
78,737
2,908
274
25,237
218
146
LSD1KORes
RCOR1/2 DKO+LSD1

Supplementary Figure 7. LSD1 re-introduction rescues gene de-repression caused by LSD1 loss in ESCs.
(a) Western blotting analysis of LSD1*Res* cells with and without DOX induction. WT and LSD1 KO cell lysates served as controls. Levels of LSD1 and HA-LSD1 in each lane were quantified by normalizing with Tubulin signals of the corresponding lane. Normalized ratios were provided under each blot. Experiments were repeated three times independently with similar results observed. Source data are provided as a Source Data file.
(b) Genome browser view of RNA-seq signals of *Rnf213* gene in LSD1 KO ESCs and LSD1*Res* cells with and without DOX treatment.
(c) Hierarchical clustering analysis of RNA-seq signals of 1,696 genes upregulated by LSD1 deletion in LSD1*Res* cells with and without DOX induction. Numbers below the heatmap denote the 2 biological replicates of each condition.
(d) GSEA analysis of 1,696 genes upregulated by LSD1 deletion comparing LSD1*Res* cells with and without DOX induction.
(e) Genome browser view of LSD1 (top) and P300 (bottom) ChIP-Rx signals at the *Rnf213* locus in LSD1*Res* cells with and without DOX induction.
(f) Heatmap analysis showing LSD1 ChIP-Rx (left) and HA ChIP-seq (right) signals in LSD1*Res* cells with and without DOX induction. Signals were centered on total LSD1 peaks and the profiles are sorted in a descending order of LSD1 occupancy in WT ESCs.
(g) Box plot analysis of LSD1 ChIP-Rx (top) and HA ChIP-seq (bottom) signals in LSD1*Res* cells with and without DOX induction at 82,923 LSD1 peaks. n=2 biologically independent experiments. Center line: median; top and bottom hinges of box: the third and first quantiles; whiskers: quartiles ± 1.5 × interquartile range. P-values were calculated using two-sided Wilcoxon signed-rank tests.
(h) Box plot analysis of P300 occupancy at 21,699 LSD1 enriched active enhancers in LSD1*Res* cells with and without DOX induction. n=2 biologically independent experiments. The three clusters are as in Fig. 2e with number of peaks in each cluster denoted. Center line: median; top and bottom hinges of box: the third and first quantiles; whiskers: quartiles ± 1.5 × interquartile range. P-values were calculated using two-sided Wilcoxon signed-rank tests.
(i) Box plot analysis of RNA-seq signals of nearest genes to LSD1 peaks at the three clusters in Fig. 2e in LSD1*Res* cells with and without DOX induction. n=2 biologically independent experiments. Center line: median; top and bottom hinges of box: the third and first quantiles; whiskers: quartiles ± 1.5 × interquartile range. P-values were calculated using two-sided Wilcoxon signed-rank tests.
(j) Venn diagrams indicating the overlap of LSD1 (left) and HA (right) ChIP-Rx peaks in LSD1*Res* and RCOR1/2 DKO+LSD1 cells upon DOX induction.
(k) Box plot analysis indicating the Log 2 fold change of LSD1 ChIP-Rx signals with and without DOX induction in LSD1*Res* and RCOR1/2 DKO+LSD1 cells. n=2 biologically independent experiments. Center line: median; top and bottom hinges of box: the third and first quantiles; whiskers: quartiles ± 1.5 × interquartile range. P-values were calculated using two-sided Wilcoxon signed-rank tests.

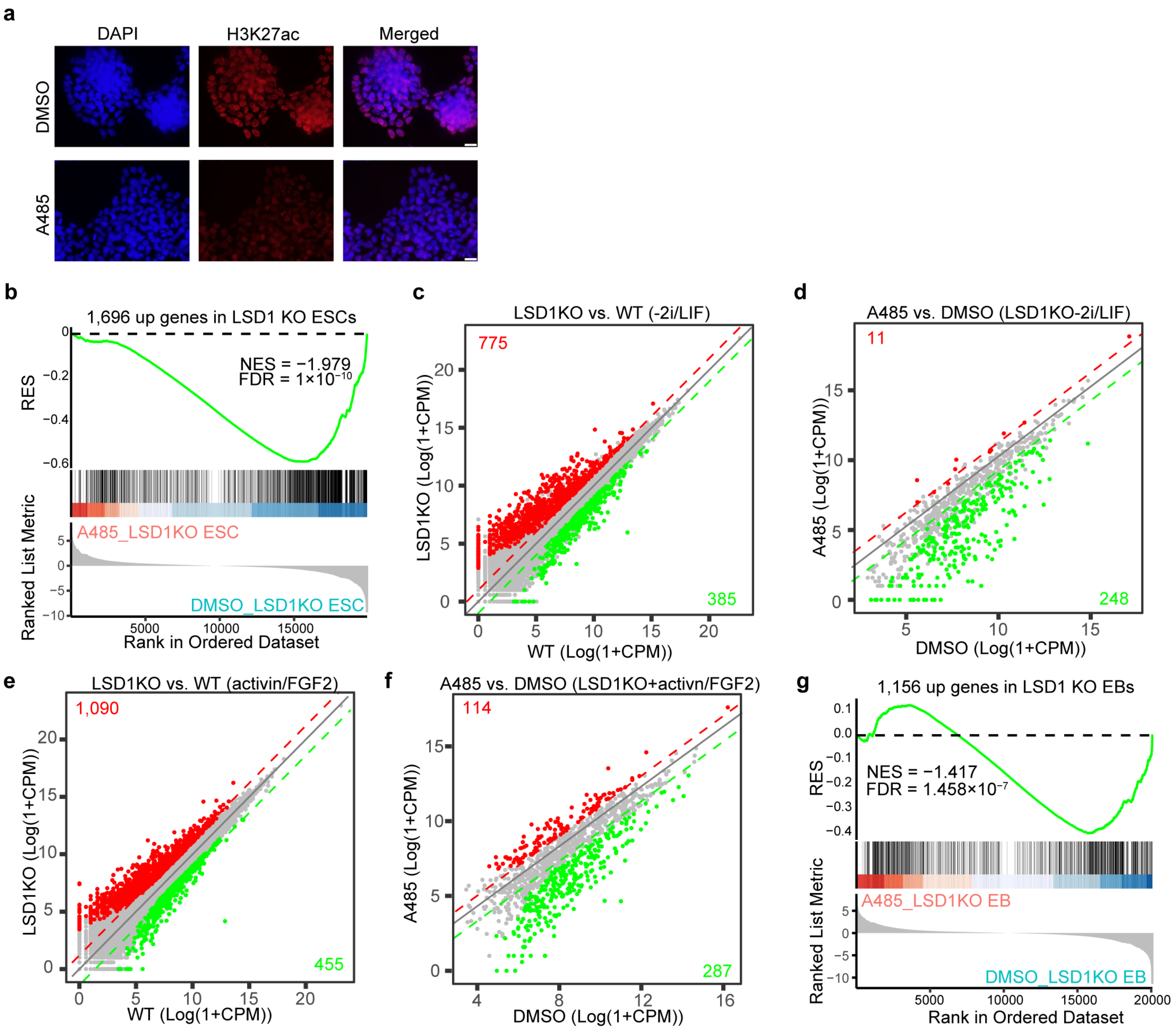

a
DAPI
H3K27ac
Merged
DMSO
A485
b
1,696 up genes in LSD1 KO ESCs
NES = −1.979
FDR = 1×10^-10
RES
Ranked List Metric
A485_LSD1KO ESC
DMSO_LSD1KO ESC
Rank in Ordered Dataset
c
LSD1KO vs. WT (-2i/LIF)
775
385
LSD1KO (Log(1+CPM))
WT (Log(1+CPM))
d
A485 vs. DMSO (LSD1KO-2i/LIF)
11
248
A485 (Log(1+CPM))
DMSO (Log(1+CPM))
e
LSD1KO vs. WT (activin/FGF2)
1,090
455
LSD1KO (Log(1+CPM))
WT (Log(1+CPM))
f
A485 vs. DMSO (LSD1KO+activn/FGF2)
114
287
A485 (Log(1+CPM))
DMSO (Log(1+CPM))
g
1,156 up genes in LSD1 KO EBs
NES = −1.417
FDR = 1.458×10^-7
RES
Ranked List Metric
A485_LSD1KO EB
DMSO_LSD1KO EB
Rank in Ordered Dataset

Supplementary Figure 8. P300/CBP inhibition rescues gene misregulation caused by LSD1 loss.

(a) Immunostaining of H3K27ac in ESCs treated with DMSO or 10μM A485 for 24 hours. Scale bar: 100μm. Experiments were repeated three times independently with similar results observed.

(b) GSEA analysis of 1,696 genes upregulated by LSD1 deletion in ESCs comparing LSD1 KO ESCs treated with 10μM A485 and DMSO.

(c) Correlation plots of RNA-seq data between LSD1 KO and WT ESCs under spontaneous differentiation conditions for two generations. Statistical significance was determined by two-sided Wald test and p-values were corrected for multiple testing using the Benjamini-Hochberg method. Significantly up- and downregulated genes are labelled in red and green with numbers of genes noted, respectively.

(d) Correlation analysis of upregulated genes in LSD1 null ESCs upon spontaneous differentiation (775 red genes in c) treated with respective A485 and DMSO. Statistical significance was determined by two-sided Wald test and p-values were corrected for multiple testing using the Benjamini-Hochberg method. Significantly up- and downregulated genes are labelled in red and green with numbers of genes noted, respectively.

(e) Correlation plots of RNA-seq data between LSD1 KO and WT ESCs under EpiLC differentiation. Statistical significance was determined by two-sided Wald test and p-values were corrected for multiple testing using the Benjamini-Hochberg method.

(f) Correlation analysis of upregulated genes in LSD1 null ESCs upon EpiLC differentiation (1,090 red genes in e) treated with respective A485 and DMSO. Statistical significance was determined by two-sided Wald test and p-values were corrected for multiple testing using the Benjamini-Hochberg method. Significantly up- and downregulated genes are labelled in red and green with numbers of genes noted, respectively.

(g) GSEA analysis of 1,156 genes upregulated by LSD1 deletion in EBs comparing LSD1 KO EBs treated with 0.4μM A485 and DMSO during differentiation.
