# Supplementary material for: Demethylase-independent roles of LSD1 in regulating enhancers and cell fate transition
Source: Nat Commun. 2023 Aug 22;14:4944. doi: 10.1038/s41467-023-40606-1 (PMC10444793; doi:10.1038/s41467-023-40606-1)
Supplement: Supplementary file 2 — Reporting Summary [file 41467_2023_40606_MOESM2_ESM.pdf]

nature portfolio

Corresponding author(s): Kaixiang Cao

Last updated by author(s): Jul 6, 2023

Reporting Summary

Nature Portfolio wishes to improve the reproducibility of the work that we publish. This form provides structure for consistency and transparency in reporting. For further information on Nature Portfolio policies, see our Editorial Policies and the Editorial Policy Checklist.

Statistics

For all statistical analyses, confirm that the following items are present in the figure legend, table legend, main text, or Methods section.

n/a	Confirmed	
☐	☒	The exact sample size (*n*) for each experimental group/condition, given as a discrete number and unit of measurement
☐	☒	A statement on whether measurements were taken from distinct samples or whether the same sample was measured repeatedly
☐	☒	The statistical test(s) used AND whether they are one- or two-sided<br>*Only common tests should be described solely by name; describe more complex techniques in the Methods section.*
☒	☐	A description of all covariates tested
☒	☐	A description of any assumptions or corrections, such as tests of normality and adjustment for multiple comparisons
☐	☒	A full description of the statistical parameters including central tendency (e.g. means) or other basic estimates (e.g. regression coefficient) AND variation (e.g. standard deviation) or associated estimates of uncertainty (e.g. confidence intervals)
☐	☒	For null hypothesis testing, the test statistic (e.g. *F*, *t*, *r*) with confidence intervals, effect sizes, degrees of freedom and *P* value noted<br>*Give P values as exact values whenever suitable.*
☒	☐	For Bayesian analysis, information on the choice of priors and Markov chain Monte Carlo settings
☒	☐	For hierarchical and complex designs, identification of the appropriate level for tests and full reporting of outcomes
☒	☐	Estimates of effect sizes (e.g. Cohen's *d*, Pearson's *r*), indicating how they were calculated

Our web collection on statistics for biologists contains articles on many of the points above.

Software and code

Policy information about availability of computer code

Data collection	No software was used for data collection.
Data analysis	Image J v1.53, Trim Galore v0.6.6, Bowtie v2.4.4, SAMtools v1.12, Picard v2.25.5, deepTools v3.5.1, BEDTools v2.30.0, MACS2 v2.2.7.1, ChIPseeker v1.28.3, GenomicRanges v1.46.0, BRGenomics v1.10.0, STAR v2.7.9a, DESeq2 v1.32.0, ggpubr v0.6.0, pheatmap v1.0.12, clusterProfiler v4.6.2, Cell Ranger v7.1.0, Metascape 3.5, and Seurat v4.3.0.

For manuscripts utilizing custom algorithms or software that are central to the research but not yet described in published literature, software must be made available to editors and reviewers. We strongly encourage code deposition in a community repository (e.g. GitHub). See the Nature Portfolio guidelines for submitting code & software for further information.

Data

Policy information about availability of data

All manuscripts must include a data availability statement. This statement should provide the following information, where applicable:

- Accession codes, unique identifiers, or web links for publicly available datasets
- A description of any restrictions on data availability
- For clinical datasets or third party data, please ensure that the statement adheres to our policy

The raw and processed high-throughput sequencing datasets including ChIP-Rx, RNA-seq and scRNA-seq generated in this study have been deposited to the Gene Expression Omnibus (GEO) database under the accession number GSE232255 and can be downloaded from the link below: https://www.ncbi.nlm.nih.gov/geo/query/acc.cgi?acc=GSE232255. Information about the mm9 genome assembly can be found at https://www.ncbi.nlm.nih.gov/assembly/GCF_000001635.18/.

Details on oligonucleotide sequences, antibodies, and additional reagents are listed in the Methods section. Source data are provided with this paper. All remaining data associated with this study are available within the Article and Supplementary Data.

Research involving human participants, their data, or biological material

Policy information about studies with human participants or human data. See also policy information about sex, gender (identity/presentation), and sexual orientation and race, ethnicity and racism.

Reporting on sex and gender	Not applicable
Reporting on race, ethnicity, or other socially relevant groupings	Not applicable
Population characteristics	Not applicable
Recruitment	Not applicable
Ethics oversight	Not applicable

Note that full information on the approval of the study protocol must also be provided in the manuscript.

Field-specific reporting

Please select the one below that is the best fit for your research. If you are not sure, read the appropriate sections before making your selection.

- [x] Life sciences
- [ ] Behavioural & social sciences
- [ ] Ecological, evolutionary & environmental sciences

For a reference copy of the document with all sections, see nature.com/documents/nr-reporting-summary-flat.pdf

Life sciences study design

All studies must disclose on these points even when the disclosure is negative.

Sample size	Sample size was not predetermined by any statistic methods. For all RNA-seq and ChIP-seq experiments, we have at least two biological replicates for each experiment, which is sufficient to perform statistical analysis. Detailed sample size and methods for statistical analysis are described in the Methods section and figure legends of specific figures.
Data exclusions	We did not exclude any data for the analysis.
Replication	Results of Western blotting, PCR, differentiation assays, and immunostaining analysis were from at least two or three biological replicates and described in the figure legends of corresponding figures. At least two biological replicates were used for generating next generation sequencing datasets and the subsequent statistic analyses. All attempts at replication for next generation sequencing experiments were successful.
Randomization	Cells were randomly allocated into experimental groups.
Blinding	Blinding was not utilized due to objective data collection methods using quantitative measurements that did not require subjective interpretation or judgment.

Reporting for specific materials, systems and methods

We require information from authors about some types of materials, experimental systems and methods used in many studies. Here, indicate whether each material, system or method listed is relevant to your study. If you are not sure if a list item applies to your research, read the appropriate section before selecting a response.

Materials & experimental systems

n/a	Involved in the study	
☐	☒	Antibodies
☐	☒	Eukaryotic cell lines
☒	☐	Palaeontology and archaeology
☒	☐	Animals and other organisms
☒	☐	Clinical data
☒	☐	Dual use research of concern
☒	☐	Plants

Methods

n/a	Involved in the study	
☐	☒	ChIP-seq
☒	☐	Flow cytometry
☒	☐	MRI-based neuroimaging

Antibodies

Antibodies used	The following primary antibodies were used in this study: anti-LSD1 (Abcam ab17721), anti-H3K4me1 (Cell Signaling Technology 5326), anti-H3K4me2 (Cell Signaling Technology 9725), Anti-H3K4me3 (Cell Signaling Technology 9727), anti-H3K27ac (Cell Signaling Technology 8173), anti-Tubulin (Developmental Studies Hybridoma Bank E7), anti-RCOR1 (Proteintech 27686-1-AP), anti-RCOR2 (Proteintech 23969-1-AP), anti-HDAC1 (Cell Signaling Technology 34589), anti-HDAC2 (Abcam ab7029), anti-H3 (Abcam ab1791), anti-P300 (Santa Cruz SC-48343X), anti-cTnT (Santa Cruz SC-20025), and anti-HA (Sigma H3663). The secondary antibodies used here were: donkey anti-rabbit IgG HRP (Sigma NA934V), sheep anti-mouse IgG HRP (Sigma NA931V), goat anti-mouse IgG-Alexa Fluor 488 (Life Technologies A11029), and goat anti-rabbit IgG-Alexa Fluor 594 (Life Technologies A32740).
Validation	Specificity for use of primary antibodies in Western blotting, ChIP-Rx, and immunofluorescence were either validated as described in the websites of vendors, in the previous literatures that were indicated in the manuscript, or in the current manuscript.<br><br>Anti-LSD1 antibody (Abcam ab17721) was validated by the vendor in Western blotting Western blotting, immunocytochemistry, and immunofluorescence. (https://www.abcam.com/products/primary-antibodies/kdm1lsd1-antibody-nuclear-marker-ab17721.html). We have previously validated this antibody in Western blotting and ChIP-seq in (Cao et al., 2018. Sci. Adv.). It was validated again in the current manuscript in Fig. 1 for Western blotting and in Fig. 2 for ChIP-seq.<br><br>Anti-H3K4me1 antibody (Cell Signaling Technology 5326) has been validated by the vendor in Western blotting, immunofluorescence, flow cytometry, chromatin immunoprecipitation, and CUT&RUN. (https://www.cellsignal.com/products/primary-antibodies/mono-methyl-histone-h3-lys4-d1a9-xp-rabbit-mab/5326)<br><br>Anti-H3K4me2 antibody (Cell Signaling Technology 9725) has been validated by the vendor in Western blotting, immunoprecipitation, immunohistochemistry, immunofluorescence, flow cytometry, chromatin immunoprecipitation, and CUT&RUN. (https://www.cellsignal.com/products/primary-antibodies/di-methyl-histone-h3-lys4-c64g9-rabbit-mab/9725)<br><br>Anti-H3K4me3 antibody (Cell Signaling Technology 9727) has been validated by the vendor in Western blotting, immunohistochemistry, immunofluorescence, chromatin immunoprecipitation, and peptide ELISA. (https://www.cellsignal.com/products/primary-antibodies/tri-methyl-histone-h3-lys4-antibody/9727)<br><br>Anti-H3K27ac antibody (Cell Signaling Technology 8173) has been validated by the vendor in Western blotting, immunofluorescence, flow cytometry, chromatin immunoprecipitation, CUT&RUN, and peptide ELISA. (https://www.cellsignal.com/products/primary-antibodies/acetyl-histone-h3-lys27-d5e4-xp-rabbit-mab/8173). We have validated this antibody before in (Cao et al., 2017. Genes & Dev.) and (Cao et al., 2018. Sci. Adv.).<br><br>Anti-Tubulin antibody (Developmental Studies Hybridoma Bank E7) has been validated by the vendor in Western blotting, immunoprecipitation, immunohistochemistry, and immunofluorescence. (https://dshb.biology.uiowa.edu/E7_2). We have validated this antibody before in (Cao et al., 2017. Genes & Dev.) and (Cao et al., 2018. Sci. Adv.).<br><br>Anti-RCOR1 antibody (Proteintech 27686-1-AP) has been validated by the vendor in Western blotting. (https://www.ptglab.com/products/RCOR1-Antibody-27686-1-AP.htm). We validated this antibody for Western blotting using RCOR1 KO cells in Fig. 4 of the current manuscript.<br><br>Anti-RCOR2 antibody (Proteintech 23969-1-AP) has been validated by the vendor in Western blotting and immunohistochemistry (to https://www.ptglab.com/products/RCOR2-Antibody-23969-1-AP.htm). We validated this antibody for Western blotting using RCOR2 KO cells in Fig. 4 of the current manuscript.<br><br>Anti-HDAC1 antibody (Cell Signaling Technology 34589) has been validated by the vendor in Western blotting, immunoprecipitation, immunofluorescence, chromatin immunoprecipitation, and CUT&RUN. (https://www.cellsignal.com/products/primary-antibodies/hdac1-d5c6u-xp-rabbit-mab/34589)<br><br>Anti-HDAC2 antibody (Abcam ab7029) has been validated by the vendor in immunoprecipitation, immunocytochemistry, and immunofluorescence. (https://www.abcam.com/products/primary-antibodies/hdac2-antibody-ab7029.html)<br><br>Anti-H3 antibody (Abcam ab1791) has been validated by the vendor in Western blotting, immunoprecipitation, immunohistochemistry, chromatin immunoprecipitation, immunocytochemistry, and immunofluorescence. (https://www.abcam.com/products/primary-antibodies/histone-h3-antibody-nuclear-marker-and-chip-grade-ab1791.html). We have validated this antibody before in (Cao et al., 2013. PLoS Gene.).<br><br>Anti-cTnT antibody (Santa Cruz SC-20025) has been validated by the vendor in Western blotting, immunohistochemistry, and immunofluorescence. (https://www.scbt.com/p/troponin-t-c-antibody-ct3)<br><br>Anti-HA (Sigma H3663) has been validated by the vendor in Western blotting, immunocytochemistry, and immunoprecipitation. (https://www.sigmaaldrich.com/US/en/product/sigma/h3663)

Eukaryotic cell lines

Policy information about cell lines and Sex and Gender in Research

Cell line source(s)	V6.5 ESC line is a gift from Rudolf Jaenisch lab. HEK293T cells were purchased from ATCC (https://www.atcc.org/products/crl-3216)
Authentication	V6.5 ESCs was authenticated genetically by the Jaenisch lab and we indirectly verified their identity by their morphology, growth behavior, and transcriptomic profiles. HEK293T cells were autheticated by ATCC and we indirectly verified their identity by the morphology and growth behavior of the cells. All genetically modified cell lines were validated by PCR, Sanger sequencing, Western blotting, and next generation sequencing.
Mycoplasma contamination	All cell lines were tested for mycoplasma contamination before the experiments. None of these cell lines are contaminated by mycoplasma.
Commonly misidentified lines (See ICLAC register)	No commonly misidentified lines was used.

Plants

Seed stocks	*Report on the source of all seed stocks or other plant material used. If applicable, state the seed stock centre and catalogue number. If plant specimens were collected from the field, describe the collection location, date and sampling procedures.*
Novel plant genotypes	*Describe the methods by which all novel plant genotypes were produced. This includes those generated by transgenic approaches, gene editing, chemical/radiation-based mutagenesis and hybridization. For transgenic lines, describe the transformation method, the number of independent lines analyzed and the generation upon which experiments were performed. For gene-edited lines, describe the editor used, the endogenous sequence targeted for editing, the targeting guide RNA sequence (if applicable) and how the editor was applied.*
Authentication	*Describe any authentication procedures for each seed stock used or novel genotype generated. Describe any experiments used to assess the effect of a mutation and, where applicable, how potential secondary effects (e.g. second site T-DNA insertions, mosiacism, off-target gene editing) were examined.*

ChIP-seq

Data deposition

☒ Confirm that both raw and final processed data have been deposited in a public database such as GEO.

☒ Confirm that you have deposited or provided access to graph files (e.g. BED files) for the called peaks.

Data access links
May remain private before publication.

https://www.ncbi.nlm.nih.gov/geo/query/acc.cgi?acc=GSE232255

Files in database submission

DKOLSD1_DOX_HA.bw
DKOLSD1_DOX_HA_R1.fastq.gz
DKOLSD1_DOX_HA_R2.fastq.gz
DKOLSD1_DOX_input.bw
DKOLSD1_DOX_input_R1.fastq.gz
DKOLSD1_DOX_input_R2.fastq.gz
DKOLSD1_DOX_LSD1.bw
DKOLSD1_DOX_LSD1_R1.fastq.gz
DKOLSD1_DOX_LSD1_R2.fastq.gz
DKOLSD1_noDOX_HA.bw
DKOLSD1_noDOX_HA_R1.fastq.gz
DKOLSD1_noDOX_HA_R2.fastq.gz
DKOLSD1_noDOX_input.bw
DKOLSD1_noDOX_input_R1.fastq.gz
DKOLSD1_noDOX_input_R2.fastq.gz
DKOLSD1_noDOX_LSD1.bw
DKOLSD1_noDOX_LSD1_R1.fastq.gz
DKOLSD1_noDOX_LSD1_R2.fastq.gz
LSD1CI_H3K27ac_1.bw
LSD1CI_H3K27ac_1_R1.fastq.gz
LSD1CI_H3K27ac_1_R2.fastq.gz
LSD1CI_H3K27ac_2.bw
LSD1CI_H3K27ac_2_R1.fastq.gz
LSD1CI_H3K27ac_2_R2.fastq.gz
LSD1CI_H3K4me1_1.bw
LSD1CI_H3K4me1_1_R1.fastq.gz

LSD1CI_H3K4me1_1_R2.fastq.gz
LSD1CI_H3K4me1_2.bw
LSD1CI_H3K4me1_2_R1.fastq.gz
LSD1CI_H3K4me1_2_R2.fastq.gz
LSD1CI_H3K4me2.bw
LSD1CI_H3K4me2_R1.fastq.gz
LSD1CI_H3K4me2_R2.fastq.gz
LSD1CI_H3K4me3.bw
LSD1CI_H3K4me3_R1.fastq.gz
LSD1CI_H3K4me3_R2.fastq.gz
LSD1CI_input_1.bw
LSD1CI_input_1_R1.fastq.gz
LSD1CI_input_1_R2.fastq.gz
LSD1CI_input_2.bw
LSD1CI_input_2_R1.fastq.gz
LSD1CI_input_2_R2.fastq.gz
LSD1KO_A485_H3K27ac.bw
LSD1KO_A485_H3K27ac_R1.fastq.gz
LSD1KO_A485_H3K27ac_R2.fastq.gz
LSD1KO_A485_input.bw
LSD1KO_A485_input_R1.fastq.gz
LSD1KO_A485_input_R2.fastq.gz
LSD1KO_DMSO_H3K27ac.bw
LSD1KO_DMSO_H3K27ac_R1.fastq.gz
LSD1KO_DMSO_H3K27ac_R2.fastq.gz
LSD1KO_DMSO_input.bw
LSD1KO_DMSO_input_R1.fastq.gz
LSD1KO_DMSO_input_R2.fastq.gz
LSD1KO_EB_H3K27ac.bw
LSD1KO_EB_H3K27ac_R1.fastq.gz
LSD1KO_EB_H3K27ac_R2.fastq.gz
LSD1KO_EB_H3K4me1.bw
LSD1KO_EB_H3K4me1_R1.fastq.gz
LSD1KO_EB_H3K4me1_R2.fastq.gz
LSD1KO_EB_input.bw
LSD1KO_EB_input_R1.fastq.gz
LSD1KO_EB_input_R2.fastq.gz
LSD1KO_H3K27ac_1.bw
LSD1KO_H3K27ac_1_R1.fastq.gz
LSD1KO_H3K27ac_1_R2.fastq.gz
LSD1KO_H3K27ac_2.bw
LSD1KO_H3K27ac_2_R1.fastq.gz
LSD1KO_H3K27ac_2_R2.fastq.gz
LSD1KO_H3K4me1_1.bw
LSD1KO_H3K4me1_1_R1.fastq.gz
LSD1KO_H3K4me1_1_R2.fastq.gz
LSD1KO_H3K4me1_2.bw
LSD1KO_H3K4me1_2_R1.fastq.gz
LSD1KO_H3K4me1_2_R2.fastq.gz
LSD1KO_H3K4me2.bw
LSD1KO_H3K4me2_R1.fastq.gz
LSD1KO_H3K4me2_R2.fastq.gz
LSD1KO_H3K4me3.bw
LSD1KO_H3K4me3_R1.fastq.gz
LSD1KO_H3K4me3_R2.fastq.gz
LSD1KO_HDAC1.bw
LSD1KO_HDAC1_R1.fastq.gz
LSD1KO_HDAC1_R2.fastq.gz
LSD1KO_HDAC2.bw
LSD1KO_HDAC2_R1.fastq.gz
LSD1KO_HDAC2_R2.fastq.gz
LSD1KO_input_1.bw
LSD1KO_input_1_R1.fastq.gz
LSD1KO_input_1_R2.fastq.gz
LSD1KO_input_2.bw
LSD1KO_input_2_R1.fastq.gz
LSD1KO_input_2_R2.fastq.gz
LSD1KO_input_3.bw

LSD1KO_input_3_R1.fastq.gz
LSD1KO_input_3_R2.fastq.gz
LSD1KO_P300.bw
LSD1KO_P300_R1.fastq.gz
LSD1KO_P300_R2.fastq.gz
LSD1KO_RCOR2.bw
LSD1KO_RCOR2_R1.fastq.gz
LSD1KO_RCOR2_R2.fastq.gz
LSD1Res_DOX_HA.bw
LSD1Res_DOX_HA_R1.fastq.gz
LSD1Res_DOX_HA_R2.fastq.gz
LSD1Res_DOX_input.bw
LSD1Res_DOX_input_R1.fastq.gz
LSD1Res_DOX_input_R2.fastq.gz
LSD1Res_DOX_LSD1.bw
LSD1Res_DOX_LSD1_R1.fastq.gz
LSD1Res_DOX_LSD1_R2.fastq.gz
LSD1Res_DOX_P300.bw
LSD1Res_DOX_P300_R1.fastq.gz
LSD1Res_DOX_P300_R2.fastq.gz
LSD1Res_noDOX_HA.bw
LSD1Res_noDOX_HA_R1.fastq.gz
LSD1Res_noDOX_HA_R2.fastq.gz
LSD1Res_noDOX_input.bw
LSD1Res_noDOX_input_R1.fastq.gz
LSD1Res_noDOX_input_R2.fastq.gz
LSD1Res_noDOX_LSD1.bw
LSD1Res_noDOX_LSD1_R1.fastq.gz
LSD1Res_noDOX_LSD1_R2.fastq.gz
LSD1Res_noDOX_P300.bw
LSD1Res_noDOX_P300_R1.fastq.gz
LSD1Res_noDOX_P300_R2.fastq.gz
WT_EB_H3K27ac.bw
WT_EB_H3K27ac_R1.fastq.gz
WT_EB_H3K27ac_R2.fastq.gz
WT_EB_H3K4me1.bw
WT_EB_H3K4me1_R1.fastq.gz
WT_EB_H3K4me1_R2.fastq.gz
WT_EB_input.bw
WT_EB_input_R1.fastq.gz
WT_EB_input_R2.fastq.gz
WT_H3K27ac_1.bw
WT_H3K27ac_1_R1.fastq.gz
WT_H3K27ac_1_R2.fastq.gz
WT_H3K27ac_2.bw
WT_H3K27ac_2_R1.fastq.gz
WT_H3K27ac_2_R2.fastq.gz
WT_H3K27ac_3.bw
WT_H3K27ac_3_R1.fastq.gz
WT_H3K27ac_3_R2.fastq.gz
WT_H3K4me1_1.bw
WT_H3K4me1_1_R1.fastq.gz
WT_H3K4me1_1_R2.fastq.gz
WT_H3K4me1_2.bw
WT_H3K4me1_2_R1.fastq.gz
WT_H3K4me1_2_R2.fastq.gz
WT_H3K4me1_3.bw
WT_H3K4me1_3_R1.fastq.gz
WT_H3K4me1_3_R2.fastq.gz
WT_H3K4me2.bw
WT_H3K4me2_R1.fastq.gz
WT_H3K4me2_R2.fastq.gz
WT_H3K4me3.bw
WT_H3K4me3_R1.fastq.gz
WT_H3K4me3_R2.fastq.gz
WT_HDAC1.bw
WT_HDAC1_R1.fastq.gz
WT_HDAC1_R2.fastq.gz

WT_HDAC2.bw
WT_HDAC2_R1.fastq.gz
WT_HDAC2_R2.fastq.gz
WT_input_1.bw
WT_input_1_R1.fastq.gz
WT_input_1_R2.fastq.gz
WT_input_2.bw
WT_input_2_R1.fastq.gz
WT_input_2_R2.fastq.gz
WT_input_3.bw
WT_input_3_R1.fastq.gz
WT_input_3_R2.fastq.gz
WT_input_4.bw
WT_input_4_R1.fastq.gz
WT_input_4_R2.fastq.gz
WT_P300.bw
WT_P300_R1.fastq.gz
WT_P300_R2.fastq.gz
WT_RCOR2.bw
WT_RCOR2_R1.fastq.gz
WT_RCOR2_R2.fastq.gz
DKOLSD1_DOX_1.count.txt
DKOLSD1_DOX_1_R1.fastq.gz
DKOLSD1_DOX_1_R2.fastq.gz
DKOLSD1_DOX_2.count.txt
DKOLSD1_DOX_2_R1.fastq.gz
DKOLSD1_DOX_2_R2.fastq.gz
DKOLSD1_DOX_3.count.txt
DKOLSD1_DOX_3_R1.fastq.gz
DKOLSD1_DOX_3_R2.fastq.gz
DKOLSD1_DOX_4.count.txt
DKOLSD1_DOX_4_R1.fastq.gz
DKOLSD1_DOX_4_R2.fastq.gz
DKOLSD1_noDOX_1.count.txt
DKOLSD1_noDOX_1_R1.fastq.gz
DKOLSD1_noDOX_1_R2.fastq.gz
DKOLSD1_noDOX_2.count.txt
DKOLSD1_noDOX_2_R1.fastq.gz
DKOLSD1_noDOX_2_R2.fastq.gz
DKOLSD1_noDOX_3.count.txt
DKOLSD1_noDOX_3_R1.fastq.gz
DKOLSD1_noDOX_3_R2.fastq.gz
DKOLSD1_noDOX_4.count.txt
DKOLSD1_noDOX_4_R1.fastq.gz
DKOLSD1_noDOX_4_R2.fastq.gz
LSD1CI_DMSO_EpiLC_RNA_1.count.txt
LSD1CI_DMSO_EpiLC_RNA_1_R1.fastq.gz
LSD1CI_DMSO_EpiLC_RNA_1_R2.fastq.gz
LSD1CI_DMSO_EpiLC_RNA_2.count.txt
LSD1CI_DMSO_EpiLC_RNA_2_R1.fastq.gz
LSD1CI_DMSO_EpiLC_RNA_2_R2.fastq.gz
LSD1CI_DMSO_FBS_RNA_1.count.txt
LSD1CI_DMSO_FBS_RNA_1_R1.fastq.gz
LSD1CI_DMSO_FBS_RNA_1_R2.fastq.gz
LSD1CI_DMSO_FBS_RNA_2.count.txt
LSD1CI_DMSO_FBS_RNA_2_R1.fastq.gz
LSD1CI_DMSO_FBS_RNA_2_R2.fastq.gz
LSD1CIEB_RNA_1.count.txt
LSD1CIEB_RNA_1_R1.fastq.gz
LSD1CIEB_RNA_1_R2.fastq.gz
LSD1CIEB_RNA_2.count.txt
LSD1CIEB_RNA_2_R1.fastq.gz
LSD1CIEB_RNA_2_R2.fastq.gz
LSD1CIEB_RNA_3.count.txt
LSD1CIEB_RNA_3_R1.fastq.gz
LSD1CIEB_RNA_3_R2.fastq.gz
LSD1CIEB_RNA_4.count.txt
LSD1CIEB_RNA_4_R1.fastq.gz

LSD1CIEB_RNA_4_R2.fastq.gz
LSD1CI_RNA_1.count.txt
LSD1CI_RNA_1_R1.fastq.gz
LSD1CI_RNA_1_R2.fastq.gz
LSD1CI_RNA_2.count.txt
LSD1CI_RNA_2_R1.fastq.gz
LSD1CI_RNA_2_R2.fastq.gz
LSD1CI_RNA_3.count.txt
LSD1CI_RNA_3_R1.fastq.gz
LSD1CI_RNA_3_R2.fastq.gz
LSD1CI_RNA_4.count.txt
LSD1CI_RNA_4_R1.fastq.gz
LSD1CI_RNA_4_R2.fastq.gz
LSD1KO_A485_EpiLC_RNA_1.count.txt
LSD1KO_A485_EpiLC_RNA_1_R1.fastq.gz
LSD1KO_A485_EpiLC_RNA_1_R2.fastq.gz
LSD1KO_A485_EpiLC_RNA_2.count.txt
LSD1KO_A485_EpiLC_RNA_2_R1.fastq.gz
LSD1KO_A485_EpiLC_RNA_2_R2.fastq.gz
LSD1KO_A485_FBS_RNA_1.count.txt
LSD1KO_A485_FBS_RNA_1_R1.fastq.gz
LSD1KO_A485_FBS_RNA_1_R2.fastq.gz
LSD1KO_A485_FBS_RNA_2.count.txt
LSD1KO_A485_FBS_RNA_2_R1.fastq.gz
LSD1KO_A485_FBS_RNA_2_R2.fastq.gz
LSD1KO_A485_RNA_1.count.txt
LSD1KO_A485_RNA_1_R1.fastq.gz
LSD1KO_A485_RNA_1_R2.fastq.gz
LSD1KO_A485_RNA_2.count.txt
LSD1KO_A485_RNA_2_R1.fastq.gz
LSD1KO_A485_RNA_2_R2.fastq.gz
LSD1KO_DMSO_EpiLC_RNA_1.count.txt
LSD1KO_DMSO_EpiLC_RNA_1_R1.fastq.gz
LSD1KO_DMSO_EpiLC_RNA_1_R2.fastq.gz
LSD1KO_DMSO_EpiLC_RNA_2.count.txt
LSD1KO_DMSO_EpiLC_RNA_2_R1.fastq.gz
LSD1KO_DMSO_EpiLC_RNA_2_R2.fastq.gz
LSD1KO_DMSO_FBS_RNA_1.count.txt
LSD1KO_DMSO_FBS_RNA_1_R1.fastq.gz
LSD1KO_DMSO_FBS_RNA_1_R2.fastq.gz
LSD1KO_DMSO_FBS_RNA_2.count.txt
LSD1KO_DMSO_FBS_RNA_2_R1.fastq.gz
LSD1KO_DMSO_FBS_RNA_2_R2.fastq.gz
LSD1KO_DMSO_RNA_1.count.txt
LSD1KO_DMSO_RNA_1_R1.fastq.gz
LSD1KO_DMSO_RNA_1_R2.fastq.gz
LSD1KO_DMSO_RNA_2.count.txt
LSD1KO_DMSO_RNA_2_R1.fastq.gz
LSD1KO_DMSO_RNA_2_R2.fastq.gz
LSD1KO_DOX_1.count.txt
LSD1KO_DOX_1_R1.fastq.gz
LSD1KO_DOX_1_R2.fastq.gz
LSD1KO_DOX_2.count.txt
LSD1KO_DOX_2_R1.fastq.gz
LSD1KO_DOX_2_R2.fastq.gz
LSD1KO_EB_A485_RNA_1.count.txt
LSD1KO_EB_A485_RNA_1_R1.fastq.gz
LSD1KO_EB_A485_RNA_1_R2.fastq.gz
LSD1KO_EB_A485_RNA_2.count.txt
LSD1KO_EB_A485_RNA_2_R1.fastq.gz
LSD1KO_EB_A485_RNA_2_R2.fastq.gz
LSD1KO_EB_DMSO_RNA_1.count.txt
LSD1KO_EB_DMSO_RNA_1_R1.fastq.gz
LSD1KO_EB_DMSO_RNA_1_R2.fastq.gz
LSD1KO_EB_DMSO_RNA_2.count.txt
LSD1KO_EB_DMSO_RNA_2_R1.fastq.gz
LSD1KO_EB_DMSO_RNA_2_R2.fastq.gz
LSD1KOEB_RNA_1.count.txt

LSD1KOEB_RNA_1_R1.fastq.gz
LSD1KOEB_RNA_1_R2.fastq.gz
LSD1KOEB_RNA_2.count.txt
LSD1KOEB_RNA_2_R1.fastq.gz
LSD1KOEB_RNA_2_R2.fastq.gz
LSD1KO_noDOX_1.count.txt
LSD1KO_noDOX_1_R1.fastq.gz
LSD1KO_noDOX_1_R2.fastq.gz
LSD1KO_noDOX_2.count.txt
LSD1KO_noDOX_2_R1.fastq.gz
LSD1KO_noDOX_2_R2.fastq.gz
LSD1KO_RNA_1.count.txt
LSD1KO_RNA_1_R1.fastq.gz
LSD1KO_RNA_1_R2.fastq.gz
LSD1KO_RNA_2.count.txt
LSD1KO_RNA_2_R1.fastq.gz
LSD1KO_RNA_2_R2.fastq.gz
LSD1KO_RNA_3.count.txt
LSD1KO_RNA_3_R1.fastq.gz
LSD1KO_RNA_3_R2.fastq.gz
LSD1KO_RNA_4.count.txt
LSD1KO_RNA_4_R1.fastq.gz
LSD1KO_RNA_4_R2.fastq.gz
LSD1KO_RNA_5.count.txt
LSD1KO_RNA_5_R1.fastq.gz
LSD1KO_RNA_5_R2.fastq.gz
LSD1KO_RNA_6.count.txt
LSD1KO_RNA_6_R1.fastq.gz
LSD1KO_RNA_6_R2.fastq.gz
LSD1Res_DOX_1.count.txt
LSD1Res_DOX_1_R1.fastq.gz
LSD1Res_DOX_1_R2.fastq.gz
LSD1Res_DOX_2.count.txt
LSD1Res_DOX_2_R1.fastq.gz
LSD1Res_DOX_2_R2.fastq.gz
LSD1Res_DOX_3.count.txt
LSD1Res_DOX_3_R1.fastq.gz
LSD1Res_DOX_3_R2.fastq.gz
LSD1Res_DOX_4.count.txt
LSD1Res_DOX_4_R1.fastq.gz
LSD1Res_DOX_4_R2.fastq.gz
LSD1Res_noDOX_1.count.txt
LSD1Res_noDOX_1_R1.fastq.gz
LSD1Res_noDOX_1_R2.fastq.gz
LSD1Res_noDOX_2.count.txt
LSD1Res_noDOX_2_R1.fastq.gz
LSD1Res_noDOX_2_R2.fastq.gz
LSD1Res_noDOX_3.count.txt
LSD1Res_noDOX_3_R1.fastq.gz
LSD1Res_noDOX_3_R2.fastq.gz
LSD1Res_noDOX_4.count.txt
LSD1Res_noDOX_4_R1.fastq.gz
LSD1Res_noDOX_4_R2.fastq.gz
MLL4KOLSD1CI_RNA_1.count.txt
MLL4KOLSD1CI_RNA_1_R1.fastq.gz
MLL4KOLSD1CI_RNA_1_R2.fastq.gz
MLL4KOLSD1CI_RNA_2.count.txt
MLL4KOLSD1CI_RNA_2_R1.fastq.gz
MLL4KOLSD1CI_RNA_2_R2.fastq.gz
MLL4KOLSD1CI_RNA_3.count.txt
MLL4KOLSD1CI_RNA_3_R1.fastq.gz
MLL4KOLSD1CI_RNA_3_R2.fastq.gz
MLL4KOLSD1CI_RNA_4.count.txt
MLL4KOLSD1CI_RNA_4_R1.fastq.gz
MLL4KOLSD1CI_RNA_4_R2.fastq.gz
MLL4KO_RNA_1.count.txt
MLL4KO_RNA_1_R1.fastq.gz
MLL4KO_RNA_1_R2.fastq.gz

MLL4KO_RNA_2.count.txt
MLL4KO_RNA_2_R1.fastq.gz
MLL4KO_RNA_2_R2.fastq.gz
MLL4LSD1DKO_RNA_1.count.txt
MLL4LSD1DKO_RNA_1_R1.fastq.gz
MLL4LSD1DKO_RNA_1_R2.fastq.gz
MLL4LSD1DKO_RNA_2.count.txt
MLL4LSD1DKO_RNA_2_R1.fastq.gz
MLL4LSD1DKO_RNA_2_R2.fastq.gz
MLL4LSD1DKO_RNA_3.count.txt
MLL4LSD1DKO_RNA_3_R1.fastq.gz
MLL4LSD1DKO_RNA_3_R2.fastq.gz
MLL4LSD1DKO_RNA_4.count.txt
MLL4LSD1DKO_RNA_4_R1.fastq.gz
MLL4LSD1DKO_RNA_4_R2.fastq.gz
RCOR1KO_EB_RNA_1.count.txt
RCOR1KO_EB_RNA_1_R1.fastq.gz
RCOR1KO_EB_RNA_1_R2.fastq.gz
RCOR1KO_EB_RNA_2.count.txt
RCOR1KO_EB_RNA_2_R1.fastq.gz
RCOR1KO_EB_RNA_2_R2.fastq.gz
RCOR1KO_EB_RNA_3.count.txt
RCOR1KO_EB_RNA_3_R1.fastq.gz
RCOR1KO_EB_RNA_3_R2.fastq.gz
RCOR1KO_EB_RNA_4.count.txt
RCOR1KO_EB_RNA_4_R1.fastq.gz
RCOR1KO_EB_RNA_4_R2.fastq.gz
RCOR1KO_RNA_1.count.txt
RCOR1KO_RNA_1_R1.fastq.gz
RCOR1KO_RNA_1_R2.fastq.gz
RCOR1KO_RNA_2.count.txt
RCOR1KO_RNA_2_R1.fastq.gz
RCOR1KO_RNA_2_R2.fastq.gz
RCOR1KO_RNA_3.count.txt
RCOR1KO_RNA_3_R1.fastq.gz
RCOR1KO_RNA_3_R2.fastq.gz
RCOR1KO_RNA_4.count.txt
RCOR1KO_RNA_4_R1.fastq.gz
RCOR1KO_RNA_4_R2.fastq.gz
RCOR2KO_EB_RNA_1.count.txt
RCOR2KO_EB_RNA_1_R1.fastq.gz
RCOR2KO_EB_RNA_1_R2.fastq.gz
RCOR2KO_EB_RNA_2.count.txt
RCOR2KO_EB_RNA_2_R1.fastq.gz
RCOR2KO_EB_RNA_2_R2.fastq.gz
RCOR2KO_RNA_1.count.txt
RCOR2KO_RNA_1_R1.fastq.gz
RCOR2KO_RNA_1_R2.fastq.gz
RCOR2KO_RNA_2.count.txt
RCOR2KO_RNA_2_R1.fastq.gz
RCOR2KO_RNA_2_R2.fastq.gz
RCOR2KO_RNA_3.count.txt
RCOR2KO_RNA_3_R1.fastq.gz
RCOR2KO_RNA_3_R2.fastq.gz
RCOR2KO_RNA_4.count.txt
RCOR2KO_RNA_4_R1.fastq.gz
RCOR2KO_RNA_4_R2.fastq.gz
RCORDKO_EB_RNA_1.count.txt
RCORDKO_EB_RNA_1_R1.fastq.gz
RCORDKO_EB_RNA_1_R2.fastq.gz
RCORDKO_EB_RNA_2.count.txt
RCORDKO_EB_RNA_2_R1.fastq.gz
RCORDKO_EB_RNA_2_R2.fastq.gz
RCORDKO_EB_RNA_3.count.txt
RCORDKO_EB_RNA_3_R1.fastq.gz
RCORDKO_EB_RNA_3_R2.fastq.gz
RCORDKO_EB_RNA_4.count.txt
RCORDKO_EB_RNA_4_R1.fastq.gz

RCORDKO_EB_RNA_4_R2.fastq.gz
RCORDKO_RNA_1.count.txt
RCORDKO_RNA_1_R1.fastq.gz
RCORDKO_RNA_1_R2.fastq.gz
RCORDKO_RNA_2.count.txt
RCORDKO_RNA_2_R1.fastq.gz
RCORDKO_RNA_2_R2.fastq.gz
RCORDKO_RNA_3.count.txt
RCORDKO_RNA_3_R1.fastq.gz
RCORDKO_RNA_3_R2.fastq.gz
RCORDKO_RNA_4.count.txt
RCORDKO_RNA_4_R1.fastq.gz
RCORDKO_RNA_4_R2.fastq.gz
WT_DMSO_EpiLC_RNA_1.count.txt
WT_DMSO_EpiLC_RNA_1_R1.fastq.gz
WT_DMSO_EpiLC_RNA_1_R2.fastq.gz
WT_DMSO_EpiLC_RNA_2.count.txt
WT_DMSO_EpiLC_RNA_2_R1.fastq.gz
WT_DMSO_EpiLC_RNA_2_R2.fastq.gz
WT_DMSO_FBS_RNA_1.count.txt
WT_DMSO_FBS_RNA_1_R1.fastq.gz
WT_DMSO_FBS_RNA_1_R2.fastq.gz
WT_DMSO_FBS_RNA_2.count.txt
WT_DMSO_FBS_RNA_2_R1.fastq.gz
WT_DMSO_FBS_RNA_2_R2.fastq.gz
WT_EB_DMSO_RNA_1.count.txt
WT_EB_DMSO_RNA_1_R1.fastq.gz
WT_EB_DMSO_RNA_1_R2.fastq.gz
WT_EB_DMSO_RNA_2.count.txt
WT_EB_DMSO_RNA_2_R1.fastq.gz
WT_EB_DMSO_RNA_2_R2.fastq.gz
WTEB_RNA_1.count.txt
WTEB_RNA_1_R1.fastq.gz
WTEB_RNA_1_R2.fastq.gz
WTEB_RNA_2.count.txt
WTEB_RNA_2_R1.fastq.gz
WTEB_RNA_2_R2.fastq.gz
WTEB_RNA_3.count.txt
WTEB_RNA_3_R1.fastq.gz
WTEB_RNA_3_R2.fastq.gz
WTEB_RNA_4.count.txt
WTEB_RNA_4_R1.fastq.gz
WTEB_RNA_4_R2.fastq.gz
WTEB_RNA_5.count.txt
WTEB_RNA_5_R1.fastq.gz
WTEB_RNA_5_R2.fastq.gz
WTEB_RNA_6.count.txt
WTEB_RNA_6_R1.fastq.gz
WTEB_RNA_6_R2.fastq.gz
WTEB_RNA_7.count.txt
WTEB_RNA_7_R1.fastq.gz
WTEB_RNA_7_R2.fastq.gz
WT_RNA_10.count.txt
WT_RNA_10_R1.fastq.gz
WT_RNA_10_R2.fastq.gz
WT_RNA_11.count.txt
WT_RNA_11_R1.fastq.gz
WT_RNA_11_R2.fastq.gz
WT_RNA_1.count.txt
WT_RNA_1_R1.fastq.gz
WT_RNA_1_R2.fastq.gz
WT_RNA_2.count.txt
WT_RNA_2_R1.fastq.gz
WT_RNA_2_R2.fastq.gz
WT_RNA_3.count.txt
WT_RNA_3_R1.fastq.gz
WT_RNA_3_R2.fastq.gz
WT_RNA_4.count.txt

WT_RNA_4_R1.fastq.gz
WT_RNA_4_R2.fastq.gz
WT_RNA_5.count.txt
WT_RNA_5_R1.fastq.gz
WT_RNA_5_R2.fastq.gz
WT_RNA_6.count.txt
WT_RNA_6_R1.fastq.gz
WT_RNA_6_R2.fastq.gz
WT_RNA_7.count.txt
WT_RNA_7_R1.fastq.gz
WT_RNA_7_R2.fastq.gz
WT_RNA_8.count.txt
WT_RNA_8_R1.fastq.gz
WT_RNA_8_R2.fastq.gz
WT_RNA_9.count.txt
WT_RNA_9_R1.fastq.gz
WT_RNA_9_R2.fastq.gz
LSD1KOEB_scRNA_1_barcodes.tsv.gz
LSD1KOEB_scRNA_1_features.tsv.gz
LSD1KOEB_scRNA_1_matrix.mtx.gz
LSD1KOEB_scRNA_1_R1.fastq.gz
LSD1KOEB_scRNA_1_R2.fastq.gz
LSD1KOEB_scRNA_2_barcodes.tsv.gz
LSD1KOEB_scRNA_2_features.tsv.gz
LSD1KOEB_scRNA_2_matrix.mtx.gz
LSD1KOEB_scRNA_2_R1.fastq.gz
LSD1KOEB_scRNA_2_R2.fastq.gz
WTEB_scRNA_1_barcodes.tsv.gz
WTEB_scRNA_1_features.tsv.gz
WTEB_scRNA_1_matrix.mtx.gz
WTEB_scRNA_1_R1.fastq.gz
WTEB_scRNA_1_R2.fastq.gz
WTEB_scRNA_2_barcodes.tsv.gz
WTEB_scRNA_2_features.tsv.gz
WTEB_scRNA_2_matrix.mtx.gz
WTEB_scRNA_2_R1.fastq.gz
WTEB_scRNA_2_R2.fastq.gz

Genome browser session
(e.g. UCSC)

Provide a link to an anonymized genome browser session for "Initial submission" and "Revised version" documents only, to enable peer review. Write "no longer applicable" for "Final submission" documents.

Methodology

Replicates

ChIP-Rx were performed with at least two biological replicates.

Sequencing depth

Samples were sequenced at least 20 million reads per end with 150bp read length on each end. Refer to GSE232255 for sequencing depth for each sample.

Antibodies

Details on Antibody information have been provided in Methods section

Peak calling parameters

Peaks were called using MACS2 v2.2.7.1 with option ‘nomodel’ and peak annotation was performed with R package ChIPseeker v1.28.3.

Data quality

Raw reads were processed with Trim Galore v0.6.6 (https://www.bioinformatics.babraham.ac.uk/projects/trim_galore/) to remove adaptors and low-quality reads with the parameter “-q 25” and then aligned to the mouse mm9 and human hg19 genome assemblies using Bowtie v2.4.4 with default parameters. All unmapped reads, low mapping quality reads (MAPQ < 30) and PCR duplicates were removed using SAMtools v1.12 78 and Picard v2.25.5 (https://broadinstitute.github.io/picard/).

Software

All unmapped reads, low mapping quality reads (MAPQ < 30) and PCR duplicates were removed using SAMtools v1.12 and Picard v2.25.5 (https://broadinstitute.github.io/picard/). The number of spike-in hg19 reads was counted with SAMtools v1.12 and normalization factor alpha = 1e6/hg19_count was calculated. Normalized bigwig was generated with bamCoverage function from deepTools v3.5.1 using scale factors calculated above and reads mapped to the ENCODE blacklist regions were removed using BEDTools v2.30.0. Peaks were called using MACS2 v2.2.7.1 with option 'nomodel' and peak annotation was performed with R package ChIPseeker v1.28.3 82,83. K-means clustering was performed and nearest-gene log changes in gene expression in the heat map of clustered peaks were generated using deepTools v3.5.1. Overlapping and unique peaks were generated using findOverlaps function from R package GenomicRanges v1.46.0 84. For occupancy boxplot representation at poised, intermediate, and active enhancers, normalized readcounts overlapping each region was calculated with getCountsByRegions function from R package BRGenomics v1.10.0 (https://mdeber.github.io) and log-transformed after adding a pseudo-count of 1. For occupancy boxplot representation from the clustered heatmaps, the matrix generated from deepTools v3.5.1 81 was used to calculate the average coverage under each genome region.
